# Depression and Post Traumatic Stress amongst female sex workers in Soweto, South Africa: A cross sectional, respondent driven sample

**DOI:** 10.1371/journal.pone.0196759

**Published:** 2018-07-05

**Authors:** Jenny Coetzee, Janice Buckley, Kennedy Otwombe, Minja Milovanovic, Glenda E. Gray, Rachel Jewkes

**Affiliations:** 1 Perinatal HIV Research Unit, Faculty of Health Sciences, University of the Witwatersrand, Chris Hani Baragwanath Academic Hospital, Johannesburg, South Africa; 2 School of Public Health, Faculty of Health Sciences, University of the Witwatersrand, Johannesburg, South Africa; 3 Department of Psychiatry, Chris Hani Baragwanath Academic Hospital, Soweto, South Africa; 4 Office of the President, South African Medical Research Council, Cape Town, South Africa; 5 Gender & Health Research Unit, South African Medical Research Council, Pretoria, South Africa; Stellenbosch University, SOUTH AFRICA

## Abstract

**Background:**

Sex workers in South Africa are exposed to high levels of violence, yet little is known about their mental health needs. This study aims to understanding the prevalence of depression and post-traumatic stress disorder (PTSD) and their risk factors amongst female sex worker (FSWs) in Soweto, South Africa.

**Methods:**

A cross-sectional, respondent-driven sampling (RDS) survey enrolled 508 FSWs. Raw and RDS adjusted data were analyzed using a chi-squared test of association and multinomial regression for risk factors associated with depression and PTSD.

**Findings:**

Symptoms of severe depression were prevalent amongst 68.7%, PTSD was 39.6%, and 32.7% suffered from comorbid PTSD and depression. Experiencing ≥3 kinds of violence increased the likelihood of comorbidity (RRR4.11, 95% CI 1.52–11.12,p = 0.005). Internalised stigma increased the likelihood of one mental health condition (RRR1.25, 95% CI 1.10–1.42,p = 0.001), higher self-esteem was associated with independent (RRR1.14, 95% CI 1.05–1.25,p = 0.002) and comorbid conditions (RRR1.17, 95% CI 1.07–1.27,p = 0.001).

**Conclusion:**

Our findings highlight the sizable burden of treatable mental health conditions among FSWs in Soweto. This was driven by multiple exposures to violence, sex work related discrimination and overall moderate levels of self-esteem masking defence mechanisms. This suggests the urgent need to design and integrate services geared to the mental health needs for this population.

## Introduction

Mental health amongst sex workers (SWs) in South Africa has infrequently been studied. This is of importance given their high exposure to violence [1–6] and that sex work in South Africa is criminalized [7, 8]. This results in high levels of discrimination, a driver of mental health concerns, being perpetrated against SWs [5]. Yet very few studies address the mental health needs of SWs in South Africa. A 2017 study of SWs attending a support group in KwaZulu Natal, South Africa (n = 155), found that 78.4% of their sample suffered from depression and anxiety, while 80% engaged in binge drinking [9]. This study aims to describe the prevalence and severity of both depression and PTSD, and their risk factors amongst FSWs in Soweto, South Africa. We hypothesize that exposure to high levels of violence, and length of time as a sex worker will be strongly associated with both depression and PTSD.

Globally, the mental health of female sex workers (FSWs) has received slightly more attention than in South Africa. A survey in India found depression rates of 39% amongst FSWs (n = 1986) [10], and in Nepal they were recorded at 82.4% (n = 210) [11]. Studies from both Australia and Israel found rates of between 17% and 31%, for PTSD and 19% and 54% for depression, respectively [12, 13]. Research from Switzerland showed that 50.3% of SWs (n = 194), suffered from some form of mental health disorder. The same study found that being a migrant SW increased the risk of mental health concerns by almost nine times, while violence increased the risk by three times[14]. A second very small study based in the United States (n = 24) found an association between anxiety, depression and self-esteem amongst women who engaged in transactional sex [15].

In sub-Saharan Africa as much as 10% of the disease burden could be attributed to neuropsychiatric disorders, in 2001 [16]. In South Africa, data from a 2002–2004 nationally representative household survey found that adult lifetime prevalence of depression was 9.7%, while past year prevalence was 4.9% [17]. Findings from a study of women in an informal settlement in South Africa showed high levels of depression amongst young women aged 18–30 years (57.9%) [18]. Research has shown that approximately 16.5% of South Africans suffer from PTSD [19]. A 2008 study in South Africa (n = 4351) highlighted the strong association between PTSD and violence. More than one third of females (34.3%) reported violence exposure resulting in a 0.9–3.2 times greater risk of PTSD when compared to non-violence exposed women [20]. Furthermore, there are high rates of PTSD and depression amongst female rape survivors (28% and 34% respectively) [20–22].

Globally, exposure to violence is associated with poor mental health [23–26]. Research has also shown the significant association between depression, PTSD and alcohol abuse [27–29]. A second study based in the United States (n = 24) found an association between anxiety-depression and self-esteem amongst women who engaged in transactional sex [15]. Furthermore, childhood exposure to violence has also been shown to impact negatively upon adult mental health [28]. Experiences of childhood trauma can cause physiological changes in the brain, potentially impacting upon emotional stability and relationship formation during adulthood, which can lead to further vulnerability to violence [30]. Past year sexual assault is associated with PTSD [29]. Level of depression is also associated with poorer adherence to medication, lower levels of education and being female [17, 31, 32]. Thus, this study aims to describe the prevalence and severity of both depression and PTSD, and their risk factors amongst FSWs in Soweto, South Africa.

## Method

Our cross-sectional study was conducted in Soweto where 508 FSWs were recruited between February and September 2016. The locale is predominantly urban and peri-urban, low-income with limited educational and employment opportunities. It has the highest population density in South Africa: an estimated two million inhabitants within >40 suburbs across 61km^2^. Eleven apartheid-era, former single-sex, and ethnically segregated hostels house an estimated 40,000 residents. In 2013, the Perinatal HIV Research Unit (PHRU) began providing HIV services to SWs within the township. Their fixed clinic was used as a base for the research [33]. Formative work included cognitive interviews (n = 12), and a pilot study (n = 40) [2, 33]. The full methodology has been extensively described in previous research [2].

Inclusion criteria for the main study were: biologically female, over 18 years, sold sex in Soweto at the time of the study, knew their recruiter, and gave voluntary informed consent to participate. In determining the sample size, a two-sided calculation was used to detect a 20% difference between HIV prevalence among FSWs who were presumed to be exposed to violence versus those FSWs presumed to be unexposed to violence. A sample size of 500 was estimated to ensure sufficient power of analysis.

A respondent-driven sampling (RDS) recruitment strategy was used [34]. The method is a popular way to recruit marginalized populations for population-level estimates [35, 36]. A total of 11 seeds (initial participants) were used during the study. Similar to a chain referral method [35], all participants (including seeds) were given three coupons with which to recruit potential participants. Seeds and subsequently enrolled participants were asked to give the coupons to randomly selected women they knew and who knew them, who, like themselves, sold sex in Soweto, and who were older than 18 years. Recruitment chains were mapped between each seed and all subsequent recruits[36, 37].

After screening, participants gave their written consent to participate in the study, and then completed a 45-minute, interviewer-administered questionnaire in English, isiZulu or seSotho. Participants were then given a maximum of three uniquely coded coupons and reimbursed R100·00 (~$7·69, at time of study). A further R20·00 (~$1·56) secondary incentive for successful recruitment was paid 7–10 days later. Primary reimbursement and secondary incentives were increased five months into the study to cover additional costs to participate[2].

Data were captured directly onto Lenovo tablets using the REDCap electronic data management system [38], hosted by the University of the Witwatersrand, South Africa. The database housed built-in skip patterns and algorithms. Duplicate data were collected on a convenience sample of 12% of the final sample size with an error rate of 0·6%. Ethical approval was provided by the Human Research Ethics Committee (Medical) of the University of the Witwatersrand, South Africa.

## Measures

Socio-demographic characteristics were scored as single items. Questions included: date of birth, home language (one of South Africa’s 11 official languages or ‘other’), and place of birth (South African provinces or countries with a shared national border). Place of birth was used to indicate whether an individual was from Gauteng or a migrant (national/non-national). They were also questioned on their highest level of education achieved (no schooling; primary school incomplete; primary school completed; high school incomplete; high school completed; post school qualification/s, dichotomized into incomplete versus completed post-school qualifications). Previously being pregnant, having had a child die and number of children under 12 years of age were also asked.

Symptoms of depression were assessed using the 20-item CES-D scale [39]. Questions included ‘During the past week I was worried by things that usually don't worry me’, and ‘During the past week I felt I was just as good as other people’. Responses ranged from: 0 ‘rarely/none of the time’, 1 ‘some of the time (1-2days)’, 2 ‘a moderate amount of time (2–4 days)’ and 3 ‘most of the time (5–7 days)’. Scores were tallied and the scale alpha was 0.84. A cut-off score of 21 was also used to indicate a high level of depression symptoms.

The presence of post-traumatic stress symptoms was assessed using the PTSD-8[40]. Participants were asked 8 questions referring to having ‘recurrent thoughts or memories of the event’, and ‘feeling jumpy, get a fright easily’, when they thought about any event which they had found traumatic. The questions were not asked specifically about one traumatic event as this population is exposed to multiple forms of trauma on an ongoing basis [41]. Evidence has shown the increased risk and severity of PTSD for women exposed to multiple forms of trauma [42]. Responses were scored as 1 ‘not at all’ to 4 ‘most of the time’. A confirmatory factor analysis was run to confirm the three subscales (hypervigilance, avoidance, intrusion). Scores were tallied for a PTSD scale which showed good Cronbach alpha at 0.89. A second variable was then generated indicating the clinical DSM-IV classification of PTSD (a minimum score of three for each of the three subscales).

To enable a multinomial logistic regression showing depression or PTSD only, comorbid conditions, or no mental health concerns, the mental health variables were each dichotomised. A new categorical variable was then generated which showed depression or PTSD only, comorbid PTSD and depression, and no mental health concerns.

To assess suicidality the question was asked: “In the past year, have you attempted suicide?”, and to assess suicide ideation “I want to ask you a question about the past month (four weeks), has the thought of ending your life been in your mind?”, and “Within the past year I have felt suicidal because I am a sex worker?”. One variable was then generated which indicated no suicidality, ideation only, attempted suicides only, and both ideation and attempted suicide.

Internal stigma were assessed using an adaptation of the People Living with HIV Stigma Index[43] including 8 questions. Internalisation of stigma in the past year was assessed by asking if participants felt ashamed, guilty, blamed themselves, felt unworthy or punished, felt isolated, avoided social gatherings or felt suicidal because of being a SW. Questions were phrased such as: ‘Within the past year I have felt ashamed because I am a sex worker?’. Responses ranged from 1 ‘strongly agree’, to 4 ‘strongly disagree’, higher scores indicated more reported internalised stigma. Both exploratory and confirmatory factor analyses were performed to confirm that internal stigma performed as a continuous measured scale. The Cronbach’s alpha was 0.70.

Self-esteem was assessed using the 10-item Rosenberg Self-esteem Scale [44]. Items were scored as 3 strongly agree to 0 strongly disagree, with items 2,5,6, and 9 being reverse scored, and included questions such as “At times, I think I am no good at all”, and “I feel that I have a number of good qualities”. Items were scored providing an overall score (ranged from 0–30), with high scores indicating higher self-esteem. The Cronbach’s alpha was 0.73 indicating a strong reliability.

The original AUDIT-C [45] scale was used to show overall binge drinking. This validated scale (Cronbach’s alpha: 0.88) has been used in studies on FSWs [1]. Given that within the Soweto context, sex work was taking place within drinking establishments, we felt that it was important to include a question on the average volume per drink: ‘How big is a typical drink (one drink, a beer or a glass of wine etc?’ Responses reflected the volume of alcohol which had been observed by the local sex work program staff to be consumed in typical FSW venues: “no drinking, 250 mls or less (Small Beer or 1 glass of wine), 440mls, 500mls, 750 mls (Bumpie), 1 Litre, 2 Litres, 5 Litres (Big Box Wine)”. A new variable showing severe versus less severe/no binge drinking was created using the 3 original AUDIT-C items and the new volume variable. The creation of this variable has been previously detailed [2]. The new severe binge drinking variable showed good reliability (Cronbach alpha: 0.89). The original AUDIT scale had a cut-off score of ≥3, a cut-off score of ≥6 indicate frequent and severe binge drinking on the new scale. While both measures are presented in the tables, only the new severe binge drinking variable is included with any further bi- or multi-variate analyses.

Violence was assessed using the WHO violence against women questionnaire [46] adapted to ask about violence specific to various perpetrator types. This included intimate partner violence (IPV), police violence, and client physical or sexual violence. In assessing physical violence, questions included: “Within the past year did any [partner/client/police] hit you with a fist or with something else (such as a beer bottle, stick or belt) which could hurt you?” and “Within the past year did any [partner/client/police] kick, drag, beat, choke or burn you?”. For sexual violence, questions included: “Within the past year did any [partner/client/police] physically force you to have sex when you did not want to?” and “Within the past year did you have sex with any [partner/client/police] when you did not want to because you were afraid of what he might do?” All forms of violence were assessed by perpetrator category before moving on to the next perpetrator category. Questions were scored on a 4-point scale ranging from 1 “none” to 4 “many”. Three new dummy variables indicating some or no physical/sexual violence was generated for intimate partners, clients, and police were generated.

The childhood trauma questionnaire (CTQ) which measured 5 dimensions: neglect (physical and emotional) and abuse (emotional, physical and sexual) was also included [47]. Twelve questions were asked, including: “I saw or heard by mother beaten by her husband or boyfriend”, and “I had sex with someone who was not my boyfriend because I was threatened or frightened or forced”. Items were scored on a 4-point scale ranging from 1 “never” to 4 “very often”. Items were dichotomised to create a “none/rarely” and “many/very often”. In our study the CTQ yielded a Cronbach alpha of 0·77, suggesting good reliability. Data was also dichotomised to show high levels of emotional and physical abuse, and neglect. An additional question on first sex was added to show some/none sexual abuse: “which of the following statements most closely described your experiences the first time you had sexual intercourse? I was willing, persuaded, tricked, forced, or raped”. For this item, non-consensual sex was defined as being “tricked, forced, or raped”. Finally, a new variable was generated which indicated the co-occurrence of violence in the past year by each perpetrator category and by childhood abuse. This was categories to show exposure to no violence, exposure to one type of violence, to two types of violence or to three or more kinds of violence.

Coupon numbers were recorded and linked between seeds, recruiters and subsequent recruits to use RDS weights for population level estimates. Participants were asked a three-part question to obtain their network size: ‘How many FSWs do you know, who also know you, in Soweto? Of those FSWs, how many are over the age of 18 years? Of these FSWs, how many have you seen over the past month?’ The final response signified participants’ network sizes. RDS weighting used for the adjustment was based on participants’ relative network size and recruitment chains. Population-level estimates were developed using crude sample data that was adjusted to reflect the target population based upon the RDS-II weight.

## Statistical analysis

In analyzing RDS data, specialized statistical software (Respondent-Driven Sampling Analysis Tool (RDSAT)[48] and Respondent Driven Sampling Analyst (RDS-Analyst) [49]) were used to assess RDS assumptions critical to ensuring that population level estimated could be generated. These included homophily, and convergence, which have previously been described in detail for this study[50]. As neither package performs statistical testing, interpretation was based upon adjusted 95% confidence intervals (95% CI). Significance was assumed if there was no overlap between 95% CI. Univariate and bivariate descriptive statistics and frequencies were determined for categorical variables presented by mental health condition.

For inclusion within the multivariate model, variables needed to be significant at a bivariate level on interpretation of the 95% CI, or suggested by the literature to be relevant to vulnerability for mental health concerns. Both RDSAT and RDS-Analyst are currently limited in its ability to perform multivariate analysis. Thus, RDS weights were exported using the RDS-II function on RDS-Analyst for the multivariate analysis which was performed in STATA 15. Cluster analyses were performed to confirm no clustering due to recruitment. The analysis presented is a multinomial logistic regression run for both weighted and unweighted data and providing a relative risk ratio (RRR). Final models were recursively developed using backward and forward stepwise elimination in which variables were retained in the final model at p ≥ 0.05. In addition to the final variables presented in the models, variables considered for inclusion within the models were: level of education, place born, pregnancy, child death, and number of children under 12 years. The final analyses presented for both weighted and unweighted models included only variables which were then pre-selected based upon the sensitivity analyses. Minor differences existed between the weighted and unweighted models, offering very similar models in explaining factors associated with comorbid depression and PTSD. One observation was dropped across the multivariate analyses due to missing responses.

## Results

In total, 508 FSWs were enrolled into the study over 7 months during 2016. All but two participants were black African, with sixteen cross-border immigrants. Recruitment chains progressed up to a maximum of 25, with 2 seeds being highly productive (10–25 waves), 4 seeds minimally productive (4–8 waves), and 5 non-productive seeds. Equilibrium, homophily and convergence were achieved on all key variables examined, including depression, PTSD, age, education and severe binge drinking (see S1 Data).

As shown in Table 1, the median age of FSWs in Soweto was 31 (IQR: 25–37) years. Almost three quarters were born in Gauteng (71.6%) and had incomplete schooling (74.2%). Almost half (48.2%) slept at home or in non-SW related venues. The remainder slept in flats or abandoned buildings and hostels (24.6%), or SW related venues such as brothels (28.7%). Most had been pregnant in their lifetime (87.9%) with 18.1% reporting the death of a child, and the median number of children under the age of 12 years was 1.2. Almost two thirds reported that they regularly went without food (63.0%). No violence was reported by 14.4% of FSWs. One type of violence was reported by 24.3%, two types of violence by 31.0% of FSWs, and three forms of violence were reported to co-occur amongst 30.2% of FSWs. The standard AUDIT-C recorded that 84.8% of FSWs engaged in problematic drinking while severe binge drinking was found amongst 55.0% of FSWs.

**Table 1:** Demographic and risk factors of FSWs in Soweto (including RDS adjusted % and 95% CI), showing univariate analyses.

VARIABLE	OVERALL	
	N(%) or median (IQR)	Adjusted % (95% CI) or median (IQR)
**Age**	30.7(18–59)	31(25–37)
**Born**		
Gauteng	346 (68·1)	71·6(64·6–77·2)
Other	162 (31.9)	28.6(22.7–35.5)
**Education**		
Incomplete schooling	384(75·6)	74·2(69·2–79·9)
Complete Secondary/Some Tertiary	124(24·4)	25·8(20·4–30·9)
**Slept in the Past week**		
Flat-abandoned building-hostel	144(28.3)	24.6(20.1–30.2)
Family/ not SW related	218(42.9)	48.2(42.1–53.9)
SW house/brothel/hotel	146(28.7)	27.2(22.6–32.2)
**Pregnant Ever**		
No	439(86.4)	12.1(8.4–15.8)
Yes	69(13.6)	87.9(84.0–91.7)
**Child Died**		
No	417 (82.1)	81.9(76.9–86.9)
Yes	91(17.9)	18.1(13.8–23.2)
**Number of children <12 years average (range**)	1.1(0–5)	1.2(1.0–2.0)
**Regularly goes without food**		
No	182(35.8)	37.0 (31.5–43.1)
Yes	326(64.2)	63.0 (56.8–68.6)
**Childhood Trauma**	19 (12–34)	18 (16–21)
**Exposures to violence**		
None	69(13.6)	14.4(10.3–18.6)
1 type	117(23.0)	24.3(19.2–29.9)
2 types	158(31.1)	31.0(25.4–36.0)
≥3 types	164(32.3)	30.2(25.9–36.5)
**AUDIT-C**		
None/Low	79(18.5)	15.2(11.3–27.5)
High	348(81.5)	84.8(79.5–88.8)
**Severe Binge Drinking**	** **	** **
Low/moderate	230(45.3)	45.0(39.1–50.8)
Severe	278(54.7)	55.0(49.5–60.8)

Table 2 showed that the median score for depressive symptoms was 25 (IQR: 19–31), with 68.7% having high depression symptoms. The median PTSD symptom score was 18 (IQR: 10–22). PTSD diagnostic criteria were met in 39.6% of FSWs. No mental health concerns were found amongst 28.9% of FSWs, while PTSD or depression were seen amongst 38.4% of FSWs and 32.7% had comorbid conditions. The majority of FSWs (85.6%) reported no suicidal ideation or attempts in the past 12 months. Suicidal ideation (but no attempts) were reported by 3.6%, while 9.8% reported ideation and attempts (1% reported attempting suicide but without ideation). The median internalised stigma score was 14 (IQR 13–16), and for self-esteem the median score was 11 (IQR 9–13).

**Table 2:** Mental health concerns amongst FSWs in Soweto, showing univariate analysis.

VARIABLE	N(%) OR MEDIAN (IQR)	ADJUSTED % (95% CI) OR MEDIAN (IQR)
**Depression**	25 (18–31)	25 (19–31)
Depression (cut point 20/21)	336(66.4)	68.7(63.4–73.6)
None/Lower levels of depressive symptoms	170(33.6)	31.3(26.5–36.5)
**PTSD score**	18 (8–22)	18(10–22)
**PTSD diagnostic criteria met**	195 (38.4)	39.6(33.5–45.2)
**Comorbidity of PTSD & Depression**	182 (59.3)	62.0(50.3–70.3)
No mental health issues	154 (30.3)	28.9(23.9–34.6)
PTSD or Depression independently	191 (37.6)	38.4(33.2–43.7)
Comorbid PTSD & Depression	163 (32.1)	32.7(27.0–38.3)
**Suicidal**	** **	** **
None	453 (87.2)	85.6(80.6–89.9)
Ideation Only	15 (2.9)	3.6(1.5–6.1)
Attempted only	5 (1.0)	1.0(0.02–1.9)
Both ideation & attempted	45(8.9)	9.8(6.3–14.4)
**Internalized Stigma**	14(8–25)	14(13–16)
**Self Esteem**	10 (1–17)	11 (9–13)

Table 3 shows demographic characteristics and risk factors by depression or PTSD, with no significant differences found between those suffering with a depression versus those not depressed. Amongst those who were depressed, the median age was 30 and 31 years, respectively, most FSWs were from Gauteng (69.0%), and 76.2% had an incomplete high school. Of those who were depressed 23.7% had slept in flats or abandoned buildings, 47.4% with family, and 28.9% in SW related venues over the preceding week. Pregnancy was reported by 89.9% of depressed FSWs, and 20.1% reported the death of a child. The median number of children was 1.2, and 60.1% of depressed women reported regularly going without food. The co-occurrence of violence ranged from no violence exposure (12.9%), experiencing only one form of violence exposure (24.2%), two forms of violence exposures (29.5%), and three or more forms of violence exposures (33.5%). The median internalised stigma score was 14 (IQR 13–16), and 54.0% of those affected by depression engaged in severe binge drinking. Self-esteem median score amongst those who had symptoms of depression was 11 (IQR 9–13).

**Table 3:** Demographic and risk factors associated with depression & PTSD for FSWs in Soweto (including RDS adjusted % and 95% CI), showing univariate and bivariate analyses.

VARIABLE	DEPRESSION		NOT DEPRESSED		PTSD SYMPTOMS		NO PTSD SYMPTOMS	
	N (%)	Adjusted % (95% CI)	N (%)	Adjusted % (95% CI)	N (%)	Adjusted % (95% CI)	N (%)	Adjusted % (95% CI)
**Age**	31.1(30.3–31.8)	31(25.0–37.0)	29.5(28.3–30.7)	30.0(25.0–37.0)	30.6(29.5–31.6)	30.0(25.0–36.0)	30.7(29.9–31.6)	31.0(25.0–37.0)
**Born**								
Gauteng	250(67.6)	69.0(60.9–76.8)	96(70.6)	76.9(68.1–84.0)	130(66.7)	70.4(59.4–78.8)	216(69.0)	72.0(64.5–79.3)
Other	120(32.4)	31.0(23.1–39.7)	40(29.4)	23.1(15.9–32.1)	65(33.3)	29.6(20.9–40.7)	97(31.0)	28.0(21.1–35.7)
**Education**								
Incomplete schooling	292(75.5)	76.2(70.2–82.0)	90(78.9)	69.3(60.3–78.5)	149(76.4)	75.6(68.0–83.4)	235(75.1)	73.3(65.7–80.4)
Complete Secondary/Some Tertiary	78(24.5)	23.8(17.3–29.9)	46(21.1)	30.7(21.4–39.6)	78(23.6)	24.4(16.6–31.8)	78(24.9)	26.7(19.7–33.4)
**Slept in the Past week**								
Flat-abandoned building-hostel	92(27.4)	23.7(17.8–29.9)	50(29.4)	25.5(19.3–34.9)	73(26.5)	26.4(19.1–35.1)	71(30.5)	23.2(17.5–30.5)
Family/ not SW related	136(40.5)	47.4(40.1–55.0)	82(48.2)	49.9(39.4–57.9)	107(38.9)	**36.9(28.6–46.1)**	111(47.6)	**55.1(46.8–62.0)**
SW house/brothel/hotel	108(32.1)	28.9(23.2–35.1)	38(22.4)	24.6(17.0–32.2)	95(34.6)	**36.6(28.1–44.4)**	51(21.9)	**21.6(16.6–27.7)**
**Pregnant Ever**								
No	38(11.3)	10.1(5.8–15.0)	31(18.2)	16.5(10.4–23.2)	29(14.9)	13.6(7.6–20.6)	40(12.8)	11.3(7.1–20.6)
Yes	298(88.7)	89.9(85.4–94.1)	139(81.8)	83.5(76.4–89.1)	166(85.1)	86.4(79.6–92.5)	273(87.2)	88.7(84.2–92.8)
**Child Died**								
No	267(79.5)	79.9(74.2–85.1)	148(87.1)	86.4(78.9–92.5)	161(82.6)	81.8(74.5–88.5)	256(81.8)	82.1(76.1–87.2)
Yes	69(20.5)	20.1(14.8–26.1)	22(12.9)	13.6(7.1–21.0)	34(17.44)	18.2(11.6–25.4)	57(18.2)	17.9(12.8–23.9)
**Number of children <12 years**	1.2 (1.1–1.3)	1.2(1.0–2.0)	1.1(0.9–1.2)	1.1(1.0–2.0)	1.1(1.0–1.2)	1.2(1.0–2.0)	1.2(1.1–1.3)	1.2(1.0–2.0)
**Regularly goes without food**								
No	135(40.2)	39.9 (33.2–46.7)	46(27.1)	28.8 (19.4–38.8)	119(43.3)	40.5 (34.1–49.0)	63(27.0)	32.4 (22.6–40.8)
Yes	201(59.8)	60.1 (53.1–66.5)	124(72.9)	71.2 (61.2–80.5)	156(56.7)	59.5 (51.4–66.4)	170(73.0)	67.6 (58.8–77.2)
**Childhood Trauma**	18.9(18.5–19.3)	18(16–21)	19.1(18.5–19.7)	18(16–21)	18.7 (18.2–19.2)	18(16–21)	19.4(18.8–19.9)	18(16–20)
**Exposures to violence**								
None	41(12.2)	12.9(7.9–18.6)	28(16.5)	18.0(10.9–24.8)	31(11.3)	11.1(5.8–17.8)	38(16.3)	16.6(10.9–24.4)
1 type	71(21.1)	24.2(17.5–30.7)	46(27.1)	23.7(16.3–31.1)	65(23.6)	25.8(17.6–35.1)	52(22.3)	23.9(17.6–30.2)
2 types	98(29.2)	29.5(21.8–35.0)	59(34.7)	34.8(25.9–43.4)	78(28.4)	29.4(21.1–38.4)	80(34.3)	32.1(24.5–38.2)
≥3 types	126(37.5)	33.5(27.5–41.5)	37(21.8)	23.5(16.6–32.2)	101(36.7)	33.7(25.2–41.9)	63(27.0)	27.4(25.2–41.9)
**Severe Binge Drinking**	** **	** **	** **	** **	** **	** **	** **	** **
Low/moderate	174(47.0)	46.0(38.1–53.8)	55(40.4)	43.4(34.0–52.8)	96(49.2)	49.1(39.7–58.3)	134(42.8)	42.4(35.1–50.0)
Severe	196(53.0)	54.0(46.7–61.7)	81(59.6)	56.6(47.8–66.0)	99(50.8)	57.6(50.6–65.0)	179(57.2)	50.9(40.9–60.1)
**Internalized Stigma**	14.8(14.5–15.2)	14 (13–16)	14.0(13.5–14.5)	13(12–15)	14.2(13.8–14.5)	14(13–16)	14.8(14.5–15.2)	14(13–16)
**Self-esteem Score**	10.5(10.1–10.9)	11(9–13)	8.5(8.0–9.1)	8(6–12)	10.4(9.9–10.9)	11(8–13)	9.5(9.0–9.9)	11(6–13)

Similarly, Table 3 also shows little difference between most demographic factors by those suffering from PTSD. The only significant difference was between PTSD groups on other sleeping arrangements in the past week: amongst PTSD affected, 36.9% had slept with family while 36.6% slept in SW related venues, compared with 55.1% who had slept with family or non-sex work related venues. Amongst those with or without PTSD, the median age was 30 (IQR 25–36) years, 70.4% came from Gauteng, and three quarters had incomplete tertiary education (75.6%). Ever having been pregnant was reported by 86.4% of FSWs and 18.2% reported the death of a child, while 1.2 was the median number of children <12 years of age. Regularly going without food was reported by 59.5% of those with PTSD, and 26.4% reported sleeping in a flat or abandoned building. The number of violence co-experiences amongst PTSD affected FSW was none (11.1%), one type (25.8%), two types (29.4%), and three or more types (33.7%). The median internalised stigma score was 14 (IQR 13–16), and severe binge drinking was found amongst 57.6% of those suffering from PTSD. Self-esteem median score was 13 (IQR 8–13) irrespective of whether PTSD was present or not.

Table 4 shows the multinomial logistic regression comparing no mental health concerns, with either depression or PTSD independently or comorbidity. The weighted analysis shows that increasing age of first selling sex increased the likelihood of having one of the two mental health concerns (RRR 1.09, 95% CI 1.04–1.14, p = 0.001), while having experienced three or more kinds of violence increased the likelihood of comorbid conditions by four times (RRR 4.11, 95% CI 1.52–11.12, p = 0.005). Experiencing three or more kinds of violence also affected the likelihood of an independent condition by 3 times (RRR 3.00, 95% CI 1.22–7.39, p = 0.017). The internalisation of stigma increased the likelihood of having an independent mental health condition (RRR 1.25, 95% CI 1.10–1.42, p = 0.001), while higher self-esteem affected both conditions independently (RRR1.14, 95% CI 1.05–1.25, p = 0.002) and comorbid conditions (RRR 117, 95% CI 107–1.27, p = 0.001).

**Table 4:** Multinomial regression analysis of factors predicting DEPRESSION and PTSD amongst female sex workers in Soweto.

	No Mental Health	Depression or PTSD Individually					Comorbid Depression & PTSD			
		Unweighted			Weighted		Unweighted		Weighted	
	(Base outcome)	RRR (95% CI)	p-value		RRR (95% CI)	p-value	RRR (95% CI)	p-value	RRR (95% CI)	p-value
**Age first sold sex**	-	**1.04(1.01–1.08)**	**0.027**		**1.09(1.04–1.14)**	**0.001**	1.00(0.96–1.04)	0.954	1.03(0.98–1.08)	0.253
**Co-occurrence of violence**										
No violence experienced	-	(ref)	-		(ref)	-	(ref)	-	(ref)	-
1 type	-	1.53(0.72–3.23)	0.265		1.77(0.75–4.18)	0.192	0.93(0.42–2.05)	0.888	1.65(0.61–4.46)	0.322
2 type	-	1.32(0.64–2.70)	0.454		1.73(0.72–4.14)	0.250	1.29(0.62–2.68)	0.519	1.66(0.61–4.46)	0.315
>3 type	-	**2.86(1.32–6.19)**	**0.008**		**3.00(1.22–7.39)**	**0.017**	**3.61(1.66–7.85)**	**0.001**	**4.11(1.52–11.12)**	**0.005**
**Internalised Stigma Score**	-	**1.14(1.04–1.24)**	**0.003**		**1.25(1.10–1.42)**	**0.001**	0.97(0.89–1.06)	0.589	1.09(0.95–1.26)	0.215
**Self-esteem Score**	-	**1.13(1.06–1.21)**	**<0.001**		**1.14(1.05–1.25)**	**0.002**	**1.17(1.10–1.25)**	**<0.001**	**1.17(1.07–1.27)**	**0.001**

## Discussion

Our findings demonstrate the significant burden of mental illness amongst FSW. Our study adds to a small body of literature in South Africa highlighting the vulnerability of FSWs to mental health conditions, and how neglected this field of study is. Our findings show that more than two thirds of FSWs in Soweto suffer from symptoms suggestive of severe depression, and more than a third suggestive of PTSD. We also highlight the high comorbidity of PTSD in depressed symptoms amongst FSWs, conditions which have been shown to be comorbid in the general population. That 10% of the population have had either suicidal ideation and attempted suicide is of concern. In addition, we show that almost three quarters of FSWs had internalised feelings of shame and guilt surrounding their engagement in sex work which increased the risk of mental health symptoms. Overall, we found low levels of self-esteem amongst FSWs, but higher self-esteem was associated with more mental health problems. Critically, our study showed the impact of repeated exposures to violence on mental health concerns. Our findings also show the high rates of binge drinking, which is consistent with those previously shown amongst FSWs in South Africa (84.8% versus 80.0%) [1].

Our findings highlight the high prevalence of depression and PTSD amongst FSWs as compared to other populations. This is important given that FSWs are marginalised and often discriminated against because of their line of work [51, 52]. Overall there is a far higher vulnerability to mental health concerns within this subset of the population where the prevalence of PTSD were double those of the general South African adult population (39.6% vs 16.5%) [19]. The rates of PTSD in our study were higher than in studies of FSWs in Australia and Israel (31% and 17%, respectively) [12, 13]. The prevalence of depression amongst FSWs in Soweto was also higher than those documented in South Africa, with more than two thirds of FSW in Soweto suffering from depression as compared with 4.9% in the total adult population [17]. While the prevalence of depression in our study was slightly lower than that recorded amongst SWs in KwaZulu Natal (66.3% versus 78.4%) [9], we note that the KwaZulu Natal study drew from a subset of SWs who were already attending a support group which may have biased the sample. Our findings are almost 10% higher than those found amongst young women in an informal settlement in South Africa [18]. While being 10–30% higher than those documented amongst FSWs across studies [10] [12, 13] they are substantially lower than the prevalence of depression found amongst Nepalese SWs [11]. Overall, our findings highlight the need to integrate effective mental health screening and interventions within existing programs which target SWs.

Exposure to violence was most strongly associated with both depression and PTSD. Previous research has shown the vulnerability of FSWs in Soweto to high levels of violence throughout their lifetime[5], and this study confirms the impact which repeat occurrences of trauma is having on the lives of these women. We showed high levels of repeated exposures to violence across the lifetime, which strongly impacts both depression and PTSD as well as their comorbidity. When compared to rape survivors in the general population, the current study shows that PTSD and depression amongst FSWs is extremely high [20, 22, 53], as is their risk of sexual violence as shown in previous findings from the same population [5]. Previous research has shown far greater levels of poly-victimization and past year exposure to violence amongst FSWs than in the general population [5]. Interestingly, exposure to childhood violence was not directly associated with mental health complications. Previous findings have shown that the relationship between childhood violence and mental health for FSWs in Soweto, is mediated through past year exposure to violence (Coetzee et al 2017). Our findings show the need for strategies to reduce the prevalence of gender based violence (GBV) in South Africa while strengthening psychosocial support services. This will be of importance for FSWs who have limited access to mental health services and are currently the focus of intensive HIV treatment and prevention campaigns. The established link between mental health and adherence to medication [31, 32] should serve as strong encouragement for policy makers and implementers alike to ensure that a strong set of preventative interventions to stop violence exposure are implemented alongside comprehensive mental health services.

Our study found a very high rate of internalised stigma and a strong association between the how women saw themselves as sex workers which both complicated and was complicated by their mental health. Such internalised stigma, as previously found, decreases access to health services, thus increasing the risk of health complications [52]. Approaches to healthcare access which are cognizant of the mental health needs of this population, and which are more holistic in nature, may be important to meeting the overall needs of the population. The criminalisation of sex work has been documented to force SWs to conceal their identities [9], and the negative social views on SW often lead to SWs feeling isolated and to increasing levels of discrimination being enacted by family, community and public protectors such as health and policing officials [5, 54, 55]. Legislative frameworks and policies which not only contradict South Africa’s Constitution, but further the vulnerability of FSWs while stifling their mental health needs, must be revised by the South African Government.

Our study found overall low levels of self-esteem amongst FSWs. While the range of self-esteem scores is from 0 to 30, our median was 11, ranging from 9–13. This suggests that FSWs in Soweto have a low to moderate low self-esteem. Interestingly, we found a strong association between increasing levels of self-esteem, and both depression and PTSD, as shown in the final model. Our findings suggest that while FSWs in Soweto have overall low levels of self-esteem, higher levels of self-esteem may be masking greater levels of psychological distress, measured through depression and PTSD. This suggests that there are possibly psychic coping strategies being employed by FSWs which may present as a slightly higher self-esteem, and mask very severe levels of underlying mental distress. More researcher is needed to understand the complex mental health challenges which FSWs face and psychological responses.

The high levels of overall binge drinking found amongst FSWs [1], and that more than 50% of the population engage in severe and frequent binge drinking, shows that alcohol is plausibly used as a coping mechanism despite its lack of association with either mental health outcome. Further research is required in order to understand the link between mental health concerns and alcohol consumption in this population. Current SW health strategies reflect on the need for substance abuse interventions, yet South Africa has inadequate rehabilitation services for the general population, let alone a key population [55]. Furthermore, it is interesting to note that while factors such as childhood exposure to trauma, pregnancy, and education, are associated with mental ill health in the general population [17, 31, 32], they were not significantly associated with mental ill health in this population. Thus, highlighting the importance of future studies on mental ill health amongst SWs.

Our study had several limitations including that the CES-D measure did not provide a diagnostic measure of depression, but rather an indication of high levels of depressive symptoms. The same limitation applies to our measure of PTSD. The AUDIT-C short measure is possibly inadequate to assess chronic binge drinking, and despite efforts to improve the measure, results should be interpreted with caution. Future work needs to understand the defence mechanisms underpinning the overall low self-esteem in this sample, and how this is functioning in combination with other mental health concerns. The data were collected cross-sectionally. We did not ask about treatment for mental health concerns.

## Conclusions and recommendations

In South Africa, mental health is a largely neglected public health concern. Our findings highlight the burden of vulnerability for FSWs in Soweto, who are exposed to poor living and working conditions while being afflicted with a serious burden of mental health conditions. There is limited availability of mental health services in South Africa. Thus, the roll-out of sex work programs in South Africa is an opportune point from which to provide some basic screening, counselling, and supportive services. However, the pervasive nature of violence in South Africa, particularly amongst this population, and the lack of political will to address this will make sustaining mental health a challenge. Research is needed which expands on our understanding of mental health in this population, and on mechanisms to treat or prevent mental health conditions within the context of repeat exposures to violence, minimal access to resources, and a highly stigmatised profession. The lack of knowledge on these factors compromises South Africa’s ability address mental health, and the scaling up sex work programs.

## Supporting information

S1 Data (PDF)
