# Supplementary material for: Depression and Post Traumatic Stress amongst female sex workers in Soweto, South Africa: A cross sectional, respondent driven sample
Source: PLoS One. 2018 Jul 5;13(7):e0196759. doi: 10.1371/journal.pone.0196759 (PMC6033380; doi:10.1371/journal.pone.0196759)
Supplement: S1 Data — (PDF) [file pone.0196759.s001.pdf]

```
\begin{longtable}[c]{@{}llllllllllll@{}}
\toprule
\begin{quote}
age
\end{quote} & \begin{quote}
edu
\end{quote} & \begin{quote}
numbchild
\end{quote} & \begin{quote}
Ip employ
\end{quote} & \begin{quote}
Binge drink
\end{quote} & \begin{quote}
Ext stigma
\end{quote} & \begin{quote}
Int stigma
\end{quote} & \begin{quote}
Dep \& ptsd
\end{quote} & \begin{quote}
Age firstsw
\end{quote} & \begin{quote}
Nselfesteem\_score
\end{quote} & \begin{quote}
selfesteem\_score
\end{quote} & \begin{quote}
Nchild\_died
\end{quote} & \begin{quote}
venn2 forms experienced\_violence
\end{quote} & \begin{quote}
hivstatus
\end{quote}\tabularnewline
\midrule
\endhead
34 & incomplete schooling & 2 & Yes & 10 & 8 & 14 & comorbid ptsd
+dep &
32 & 9 & 9 & 0 & no violence & positive\tabularnewline
31 & incomplete schooling & 4 & No & 0 & 7 & 11 & comorbid ptsd +dep
&
28 & 13 & 13 & 1 & 3+ forms experienced & positive\tabularnewline
33 & incomplete schooling & 2 & Yes & 0 & 6 & 13 & comorbid ptsd
+dep &
31 & 7 & 10 & 1 & no violence & positive\tabularnewline
27 & incomplete schooling & 2 & Yes & 15 & 12 & 16 & no MH & 20 & 5
& 8
& 0 & 2 forms experienced & positive\tabularnewline
35 & incomplete schooling & 4 & Yes & 11 & 6 & 11 & depressed / PTSD
seperately & 33 & 7 & 10 & 0 & 2 forms experienced &
positive\tabularnewline
36 & incomplete schooling & 1 & No & 15 & 8 & 10 & no MH & 20 & 4 &
7 &
1 & 2 forms experienced & positive\tabularnewline
29 & incomplete schooling & 2 & Yes & 11 & 9 & 12 & no MH & 22 & 15
& 15
& 0 & 2 forms experienced & positive\tabularnewline
```

43 & incomplete schooling & 4 & Yes & 14 & 8 & 11 & no MH & 17 & 6 & 6 &
1 & 3+ forms experienced & positive\tabularnewline
21 & incomplete schooling & 0 & Yes & 0 & 6 & 13 & no MH & 19 & 13 & 13
& 0 & 2 forms experienced & positive\tabularnewline
37 & incomplete schooling & 2 & Yes & 12 & 8 & 14 & depressed / PTSD
seperately & 30 & 11 & 11 & 0 & 2 forms experienced forms experienced
form only & positive\tabularnewline
34 & matric/post school qualification & 2 & Yes & 0 & 14 & 12 & comorbid
ptsd +dep & 19 & 11 & 11 & 1 & 2 forms experienced & positive\tabularnewline
29 & incomplete schooling & 2 & Yes & 15 & 6 & 14 & depressed / PTSD
seperately & 24 & 12 & 12 & 0 & 2 forms experienced & positive\tabularnewline
33 & incomplete schooling & 2 & Yes & 9 & 6 & 13 & no MH & 30 & 6 & 9 &
0 & 2 forms experienced forms experienced form only & positive\tabularnewline
40 & incomplete schooling & 3 & Yes & 12 & 12 & 13 & depressed / PTSD
seperately & 36 & 9 & 12 & 0 & 3+ forms experienced & positive\tabularnewline
32 & incomplete schooling & 1 & Yes & 10 & 6 & 13 & no MH & 27 & 16 & 16
& 1 & 2 forms experienced & positive\tabularnewline
40 & incomplete schooling & 3 & No & 10 & 6 & 13 & no MH & 37 & 8 & 11 &
0 & 2 forms experienced forms experienced form only & positive\tabularnewline
42 & incomplete schooling & 5 & Yes & 12 & 10 & 16 & no MH & 31 & 5 & 4
& 0 & 3+ forms experienced & positive\tabularnewline
35 & matric/post school qualification & 1 & Yes & 4 & 8 & 15 & depressed
/ PTSD seperately & 30 & 14 & 14 & 0 & 3+ forms experienced & positive\tabularnewline
38 & incomplete schooling & 2 & Yes & 16 & 7 & 10 & no MH & 25 & 4 & 7 &
0 & 3+ forms experienced & positive\tabularnewline
44 & incomplete schooling & 2 & Yes & 14 & 9 & 15 & comorbid ptsd +dep &
39 & 12 & 12 & 0 & 3+ forms experienced & positive\tabularnewline
37 & incomplete schooling & 1 & Yes & 11 & 6 & 13 & no MH & 34 & 14 & 14
& 0 & 2 forms experienced forms experienced form only & positive\tabularnewline
26 & incomplete schooling & 1 & Yes & 0 & 7 & 16 & no MH & 25 & 4 & 7 &
1 & 2 forms experienced & positive\tabularnewline
28 & incomplete schooling & 1 & No & 11 & 7 & 13 & comorbid ptsd +dep &

23 & 11 & 10 & 0 & 3+ forms experienced & positive\tabularnewline
33 & incomplete schooling & 1 & Yes & 12 & 8 & 13 & depressed / PTSD
seperately & 21 & 2 & 5 & 0 & 3+ forms experienced &
positive\tabularnewline
33 & incomplete schooling & 1 & Yes & 6 & 6 & 13 & no MH & 29 & 12 &
12
& 0 & no violence & positive\tabularnewline
33 & incomplete schooling & 1 & Yes & 14 & 8 & 16 & depressed / PTSD
seperately & 29 & 11 & 11 & 1 & 2 forms experienced forms
experienced
form only & positive\tabularnewline
28 & matric/post school qualification & 1 & Yes & 15 & 6 & 18 & no
MH &
23 & 13 & 16 & 1 & 2 forms experienced & positive\tabularnewline
22 & incomplete schooling & 2 & Yes & 11 & 6 & 16 & no MH & 20 & 11
& 11
& 0 & 2 forms experienced & positive\tabularnewline
28 & incomplete schooling & 2 & Yes & 14 & 11 & 16 & comorbid ptsd
+dep
& 18 & 13 & 13 & 0 & 2 forms experienced forms experienced form only
&
positive\tabularnewline
18 & incomplete schooling & 3 & No & 4 & 7 & 14 & depressed / PTSD
seperately & 23 & 11 & 11 & 0 & 3+ forms experienced &
positive\tabularnewline
39 & incomplete schooling & 2 & Yes & 9 & 6 & 11 & no MH & 34 & 9 &
9 &
0 & 2 forms experienced & positive\tabularnewline
19 & matric/post school qualification & 0 & Yes & 16 & 6 & 17 & no
MH &
18 & 11 & 14 & 0 & 2 forms experienced & positive\tabularnewline
25 & incomplete schooling & 2 & Yes & 12 & 10 & 15 & comorbid ptsd
+dep
& 22 & 7 & 10 & 0 & 2 forms experienced & positive\tabularnewline
40 & incomplete schooling & 1 & Yes & 15 & 7 & 13 & depressed / PTSD
seperately & 27 & 1 & 4 & 1 & 2 forms experienced &
positive\tabularnewline
33 & incomplete schooling & 2 & Yes & 9 & 6 & 16 & depressed / PTSD
seperately & 32 & 19 & 19 & 0 & 2 forms experienced forms
experienced
form only & positive\tabularnewline
39 & incomplete schooling & 1 & Yes & 10 & 7 & 17 & depressed / PTSD
seperately & 25 & 15 & 15 & 0 & 2 forms experienced forms
experienced
form only & positive\tabularnewline
22 & incomplete schooling & 1 & Yes & 5 & 6 & 18 & no MH & 20 & 10 &
9 &
0 & 2 forms experienced forms experienced form only &
positive\tabularnewline
23 & incomplete schooling & 1 & Yes & 11 & 6 & 14 & depressed / PTSD
seperately & 22 & 13 & 13 & 0 & 2 forms experienced &
positive\tabularnewline
23 & incomplete schooling & 1 & Yes & 12 & 6 & 13 & depressed / PTSD
seperately & 19 & 12 & 12 & 0 & 2 forms experienced &

positive\tabularnewline
37 & incomplete schooling & 2 & Yes & 10 & 7 & 12 & depressed / PTSD seperately & 35 & 4 & 7 & 0 & 3+ forms experienced & positive\tabularnewline
36 & matric/post school qualification & 3 & Yes & 13 & 10 & 8 & no MH &
33 & 8 & 8 & 0 & 2 forms experienced forms experienced form only & positive\tabularnewline
37 & incomplete schooling & 2 & No & 10 & 9 & 15 & comorbid ptsd +dep &
30 & 12 & 12 & 0 & 2 forms experienced forms experienced form only & positive\tabularnewline
24 & incomplete schooling & 2 & Yes & 6 & 12 & 12 & comorbid ptsd +dep &
20 & 13 & 16 & 0 & 2 forms experienced & positive\tabularnewline
20 & incomplete schooling & 1 & Yes & 0 & 8 & 12 & comorbid ptsd +dep &
16 & 12 & 12 & 0 & 2 forms experienced forms experienced form only & positive\tabularnewline
30 & incomplete schooling & 1 & Yes & 11 & 7 & 13 & depressed / PTSD seperately & 26 & 14 & 14 & 0 & 2 forms experienced forms experienced
form only & positive\tabularnewline
37 & incomplete schooling & 1 & Yes & 12 & 6 & 13 & no MH & 35 & 11 & 11
& 0 & no violence & positive\tabularnewline
23 & incomplete schooling & 2 & Yes & 12 & 8 & 17 & depressed / PTSD seperately & 22 & 12 & 15 & 1 & 2 forms experienced forms experienced
form only & positive\tabularnewline
34 & incomplete schooling & 4 & No & 0 & 9 & 14 & depressed / PTSD seperately & 24 & 16 & 16 & 0 & 3+ forms experienced & positive\tabularnewline
36 & matric/post school qualification & 2 & Yes & 6 & 8 & 14 & depressed
/ PTSD seperately & 25 & 15 & 15 & 0 & 2 forms experienced & positive\tabularnewline
23 & incomplete schooling & 2 & Yes & 11 & 8 & 18 & depressed / PTSD seperately & 23 & 14 & 17 & 0 & no violence & positive\tabularnewline
31 & matric/post school qualification & 0 & Yes & 6 & 9 & 10 & comorbid
ptsd +dep & 28 & 2 & 5 & 1 & 3+ forms experienced & positive\tabularnewline
29 & incomplete schooling & 1 & No & 4 & 9 & 15 & comorbid ptsd +dep &
26 & 11 & 11 & 0 & 3+ forms experienced & positive\tabularnewline
28 & incomplete schooling & 3 & Yes & 11 & 6 & 12 & no MH & 27 & 6 & 9 &
0 & 2 forms experienced forms experienced form only & positive\tabularnewline
44 & incomplete schooling & 3 & No & 7 & 8 & 12 & depressed / PTSD seperately & 41 & 7 & 10 & 0 & 3+ forms experienced & positive\tabularnewline

20 & incomplete schooling & 0 & Yes & 8 & 8 & 14 & comorbid ptsd +dep &
19 & 11 & 11 & 0 & 2 forms experienced forms experienced form only & positive\tabularnewline
36 & matric/post school qualification & 2 & Yes & 9 & 6 & 13 & no MH &
18 & 12 & 12 & 0 & no violence & positive\tabularnewline
22 & incomplete schooling & 0 & Yes & 13 & 9 & 13 & depressed / PTSD seperately & 17 & 12 & 12 & 1 & 2 forms experienced & positive\tabularnewline
39 & incomplete schooling & 1 & Yes & 13 & 7 & 12 & comorbid ptsd +dep &
33 & 12 & 15 & 0 & 2 forms experienced & positive\tabularnewline
38 & matric/post school qualification & 4 & Yes & 11 & 15 & 9 & comorbid
ptsd +dep & 37 & 5 & 8 & 0 & 2 forms experienced & positive\tabularnewline
33 & matric/post school qualification & 2 & Yes & 5 & 6 & 10 & no MH &
29 & 4 & 3 & 0 & 2 forms experienced forms experienced form only & positive\tabularnewline
22 & matric/post school qualification & 0 & Yes & 7 & 11 & 19 & comorbid
ptsd +dep & 19 & 12 & 12 & 0 & 3+ forms experienced & positive\tabularnewline
32 & incomplete schooling & 1 & Yes & 0 & 8 & 19 & depressed / PTSD seperately & 31 & 16 & 13 & 0 & 3+ forms experienced & positive\tabularnewline
51 & incomplete schooling & 1 & Yes & 12 & 6 & 13 & depressed / PTSD seperately & 47 & 13 & 12 & 0 & no violence & positive\tabularnewline
31 & incomplete schooling & 2 & Yes & 9 & 6 & 19 & depressed / PTSD seperately & 26 & 15 & 18 & 0 & 2 forms experienced & positive\tabularnewline
24 & incomplete schooling & 1 & No & 11 & 11 & 13 & comorbid ptsd +dep &
18 & 10 & 13 & 0 & 3+ forms experienced & positive\tabularnewline
20 & matric/post school qualification & 1 & Yes & 16 & 15 & 8 & no MH &
17 & 8 & 8 & 0 & 2 forms experienced & positive\tabularnewline
23 & incomplete schooling & 1 & Yes & 13 & 8 & 15 & comorbid ptsd +dep &
20 & 13 & 13 & 1 & 2 forms experienced & positive\tabularnewline
22 & incomplete schooling & 0 & No & 12 & 6 & 16 & comorbid ptsd +dep &
21 & 16 & 16 & 1 & no violence & positive\tabularnewline
24 & incomplete schooling & 3 & No & 11 & 8 & 13 & depressed / PTSD seperately & 18 & 15 & 15 & 0 & no violence & positive\tabularnewline
25 & incomplete schooling & 2 & Yes & 16 & 8 & 13 & no MH & 19 & 7 & 7 &
0 & 2 forms experienced forms experienced form only & positive\tabularnewline
32 & incomplete schooling & 1 & Yes & 9 & 6 & 14 & depressed / PTSD

seperately & 28 & 11 & 11 & 0 & 3+ forms experienced &
positive\tabularnewline
36 & incomplete schooling & 2 & Yes & 11 & 6 & 13 & comorbid ptsd
+dep &
33 & 6 & 9 & 0 & 2 forms experienced & positive\tabularnewline
30 & incomplete schooling & 3 & No & 14 & 8 & 14 & depressed / PTSD
seperately & 29 & 12 & 12 & 1 & 2 forms experienced &
positive\tabularnewline
31 & incomplete schooling & 3 & Yes & 7 & 10 & 23 & depressed / PTSD
seperately & 21 & 13 & 13 & 1 & 2 forms experienced &
positive\tabularnewline
21 & incomplete schooling & 1 & Yes & 7 & 11 & 19 & depressed / PTSD
seperately & 15 & 19 & 19 & 0 & 3+ forms experienced &
positive\tabularnewline
30 & incomplete schooling & 1 & Yes & 11 & 8 & 14 & comorbid ptsd
+dep &
28 & 15 & 18 & 0 & no violence & positive\tabularnewline
26 & incomplete schooling & 0 & Yes & 6 & 7 & 16 & no MH & 25 & 5 &
8 &
0 & 2 forms experienced & positive\tabularnewline
25 & incomplete schooling & 1 & Yes & 12 & 6 & 15 & no MH & 24 & 8 &
11
& 0 & 3+ forms experienced & positive\tabularnewline
23 & matric/post school qualification & 0 & Yes & 8 & 9 & 15 &
comorbid
ptsd +dep & 22 & 4 & 7 & 0 & 3+ forms experienced &
positive\tabularnewline
27 & matric/post school qualification & 2 & Yes & 8 & 8 & 17 &
depressed
/ PTSD seperately & 26 & 11 & 14 & 1 & 2 forms experienced &
positive\tabularnewline
40 & incomplete schooling & 1 & Yes & 9 & 6 & 12 & comorbid ptsd
+dep &
30 & 10 & 9 & 0 & 2 forms experienced & positive\tabularnewline
29 & incomplete schooling & 2 & Yes & 9 & 6 & 20 & depressed / PTSD
seperately & 22 & 4 & 7 & 0 & no violence & positive\tabularnewline
35 & incomplete schooling & 3 & Yes & 12 & 6 & 13 & depressed / PTSD
seperately & 29 & 6 & 9 & 1 & 2 forms experienced forms experienced
form
only & positive\tabularnewline
32 & incomplete schooling & 4 & Yes & 12 & 6 & 13 & depressed / PTSD
seperately & 29 & 11 & 11 & 0 & 2 forms experienced forms
experienced
form only & positive\tabularnewline
31 & matric/post school qualification & 1 & Yes & 8 & 7 & 19 & no MH
&
25 & 6 & 9 & 0 & 2 forms experienced & positive\tabularnewline
30 & incomplete schooling & 1 & No & 4 & 8 & 14 & comorbid ptsd +dep
&
28 & 12 & 12 & 0 & 2 forms experienced & positive\tabularnewline
22 & incomplete schooling & 0 & Yes & 0 & 8 & 13 & comorbid ptsd
+dep &
14 & 6 & 9 & 0 & 3+ forms experienced & positive\tabularnewline
28 & matric/post school qualification & 1 & Yes & 11 & 6 & 13 & no

MH &
27 & 12 & 12 & 0 & 2 forms experienced & positive\tabularnewline
26 & incomplete schooling & 0 & Yes & 7 & 7 & 20 & depressed / PTSD
seperately & 24 & 11 & 11 & 0 & 3+ forms experienced &
positive\tabularnewline
36 & incomplete schooling & 1 & No & 13 & 6 & 12 & depressed / PTSD
seperately & 33 & 5 & 8 & 0 & 2 forms experienced forms experienced
form
only & positive\tabularnewline
21 & incomplete schooling & 2 & Yes & 9 & 6 & 12 & no MH & 19 & 7 &
7 &
1 & 2 forms experienced & positive\tabularnewline
38 & incomplete schooling & 7 & No & 13 & 7 & 13 & comorbid ptsd
+dep &
31 & 9 & 8 & 1 & 3+ forms experienced & positive\tabularnewline
40 & incomplete schooling & 3 & Yes & 14 & 11 & 14 & comorbid ptsd
+dep
& 18 & 15 & 15 & 0 & 2 forms experienced & positive\tabularnewline
25 & incomplete schooling & 1 & Yes & 5 & 8 & 12 & no MH & 20 & 6 &
9 &
0 & 2 forms experienced & positive\tabularnewline
47 & incomplete schooling & 5 & No & 10 & 6 & 13 & comorbid ptsd
+dep &
40 & 10 & 10 & 1 & 2 forms experienced & positive\tabularnewline
20 & incomplete schooling & 0 & Yes & 4 & 8 & 13 & comorbid ptsd
+dep &
17 & 13 & 13 & 0 & 3+ forms experienced & positive\tabularnewline
26 & incomplete schooling & 2 & No & 10 & 6 & 13 & depressed / PTSD
seperately & 19 & 13 & 13 & 0 & 2 forms experienced forms
experienced
form only & positive\tabularnewline
32 & incomplete schooling & 0 & Yes & 14 & 15 & 12 & comorbid ptsd
+dep
& 30 & 3 & 6 & 0 & 3+ forms experienced & positive\tabularnewline
30 & incomplete schooling & 1 & No & 13 & 7 & 15 & depressed / PTSD
seperately & 6 & 5 & 4 & 0 & 2 forms experienced &
positive\tabularnewline
28 & matric/post school qualification & 2 & No & 0 & 6 & 13 & no MH
& 25
& 5 & 8 & 0 & no violence & positive\tabularnewline
21 & incomplete schooling & 1 & Yes & 0 & 7 & 11 & depressed / PTSD
seperately & 17 & 8 & 8 & 0 & 2 forms experienced &
positive\tabularnewline
41 & incomplete schooling & 2 & No & 11 & 8 & 15 & comorbid ptsd
+dep &
40 & 11 & 11 & 0 & 2 forms experienced forms experienced form only &
positive\tabularnewline
35 & incomplete schooling & 4 & Yes & 12 & 6 & 11 & no MH & 25 & 6 &
9 &
1 & 2 forms experienced & positive\tabularnewline
39 & incomplete schooling & 2 & Yes & 10 & 6 & 15 & depressed / PTSD
seperately & 34 & 13 & 13 & 1 & 2 forms experienced forms
experienced
form only & positive\tabularnewline

32 & matric/post school qualification & 3 & Yes & 0 & 6 & 13 & no MH &
23 & 5 & 8 & 0 & 2 forms experienced & positive\tabularnewline
33 & incomplete schooling & 2 & Yes & 11 & 8 & 20 & depressed / PTSD seperately & 29 & 11 & 11 & 0 & no violence & positive\tabularnewline
39 & matric/post school qualification & 1 & Yes & 10 & 9 & 20 & no MH &
34 & 4 & 7 & 0 & 3+ forms experienced & positive\tabularnewline
45 & incomplete schooling & 4 & No & 11 & 7 & 17 & depressed / PTSD seperately & 39 & 12 & 12 & 1 & 2 forms experienced & positive\tabularnewline
24 & incomplete schooling & 1 & No & 14 & 7 & 17 & comorbid ptsd +dep &
22 & 13 & 13 & 0 & 3+ forms experienced & positive\tabularnewline
45 & incomplete schooling & 2 & No & 12 & 8 & 16 & depressed / PTSD seperately & 42 & 7 & 10 & 1 & 3+ forms experienced & positive\tabularnewline
31 & incomplete schooling & 2 & Yes & 13 & 8 & 14 & depressed / PTSD seperately & 28 & 13 & 13 & 0 & 2 forms experienced & positive\tabularnewline
26 & matric/post school qualification & 2 & No & 7 & 6 & 11 & no MH & 25
& 13 & 13 & 0 & 2 forms experienced & positive\tabularnewline
25 & matric/post school qualification & 2 & Yes & 13 & 8 & 15 & depressed / PTSD seperately & 23 & 11 & 11 & 0 & 2 forms experienced forms experienced form only & positive\tabularnewline
38 & matric/post school qualification & 4 & Yes & 11 & 8 & 13 & comorbid
ptsd +dep & 37 & 13 & 13 & 0 & 2 forms experienced forms experienced form only & positive\tabularnewline
35 & incomplete schooling & 2 & No & 0 & 8 & 13 & depressed / PTSD seperately & 35 & 5 & 8 & 0 & 2 forms experienced & positive\tabularnewline
22 & incomplete schooling & 1 & Yes & 17 & 7 & 20 & depressed / PTSD seperately & 20 & 11 & 14 & 1 & 3+ forms experienced & positive\tabularnewline
29 & incomplete schooling & 2 & Yes & 11 & 8 & 13 & comorbid ptsd +dep &
26 & 11 & 11 & 0 & 2 forms experienced forms experienced form only & positive\tabularnewline
31 & incomplete schooling & 1 & Yes & 13 & 6 & 12 & no MH & 26 & 11 & 11
& 0 & no violence & positive\tabularnewline
31 & incomplete schooling & 2 & No & 0 & 9 & 17 & comorbid ptsd +dep &
24 & 12 & 12 & 0 & 2 forms experienced & positive\tabularnewline
35 & incomplete schooling & 2 & Yes & 0 & 7 & 15 & depressed / PTSD seperately & 30 & 6 & 6 & 1 & no violence & negative\tabularnewline
26 & incomplete schooling & 1 & Yes & 13 & 17 & 18 & depressed / PTSD
seperately & 23 & 6 & 9 & 0 & 3+ forms experienced & positive\tabularnewline
26 & incomplete schooling & 1 & Yes & 6 & 7 & 14 & no MH & 24 & 4 &

7 &
1 & 2 forms experienced forms experienced form only &
negative\tabularnewline
49 & matric/post school qualification & 2 & Yes & 9 & 6 & 13 & no MH
&
44 & 8 & 11 & 0 & 2 forms experienced & negative\tabularnewline
31 & incomplete schooling & 0 & Yes & 3 & 12 & 20 & comorbid ptsd
+dep &
25 & 8 & 11 & 1 & 3+ forms experienced & positive\tabularnewline
29 & incomplete schooling & 1 & Yes & 0 & 8 & 12 & comorbid ptsd
+dep &
26 & 10 & 10 & 1 & 3+ forms experienced & positive\tabularnewline
33 & incomplete schooling & 2 & Yes & 10 & 7 & 21 & depressed / PTSD
seperately & 22 & 3 & 6 & 0 & 3+ forms experienced &
positive\tabularnewline
20 & incomplete schooling & 1 & Yes & 15 & 12 & 13 & depressed /
PTSD
seperately & 20 & 15 & 15 & 0 & 3+ forms experienced &
negative\tabularnewline
47 & incomplete schooling & 3 & Yes & 6 & 6 & 11 & comorbid ptsd
+dep &
40 & 11 & 11 & 0 & 2 forms experienced & positive\tabularnewline
41 & incomplete schooling & 4 & Yes & 12 & 8 & 16 & depressed / PTSD
seperately & 26 & 9 & 12 & 0 & no violence & positive\tabularnewline
32 & incomplete schooling & 2 & Yes & 0 & 9 & 17 & comorbid ptsd
+dep &
29 & 9 & 8 & 0 & no violence & negative\tabularnewline
21 & matric/post school qualification & 0 & No & 9 & 6 & 13 & no MH
& 20
& 15 & 15 & 0 & no violence & negative\tabularnewline
27 & incomplete schooling & 2 & Yes & 5 & 11 & 18 & depressed / PTSD
seperately & 25 & 16 & 19 & 1 & 3+ forms experienced &
positive\tabularnewline
23 & incomplete schooling & 1 & No & 0 & 6 & 12 & no MH & 21 & 6 & 9
& 0
& no violence & negative\tabularnewline
22 & incomplete schooling & 1 & Yes & 13 & 6 & 19 & depressed / PTSD
seperately & 19 & 7 & 10 & 0 & 2 forms experienced &
negative\tabularnewline
18 & incomplete schooling & 1 & No & 11 & 9 & 14 & comorbid ptsd
+dep &
16 & 16 & 16 & 0 & 2 forms experienced & negative\tabularnewline
26 & incomplete schooling & 2 & Yes & 5 & 10 & 12 & comorbid ptsd
+dep &
24 & 10 & 10 & 0 & 3+ forms experienced & negative\tabularnewline
26 & incomplete schooling & 1 & Yes & 9 & 8 & 14 & comorbid ptsd
+dep &
26 & 8 & 11 & 0 & 2 forms experienced & negative\tabularnewline
20 & matric/post school qualification & 1 & Yes & 6 & 8 & 11 &
comorbid
ptsd +dep & 16 & 10 & 10 & 0 & 2 forms experienced forms experienced
form only & negative\tabularnewline
40 & incomplete schooling & 2 & No & 0 & 8 & 15 & comorbid ptsd +dep
&

19 & 6 & 9 & 1 & 2 forms experienced & negative\tabularnewline
21 & incomplete schooling & 1 & Yes & 13 & 8 & 16 & depressed / PTSD seperately & 20 & 9 & 12 & 0 & 2 forms experienced forms experienced form only & negative\tabularnewline
23 & incomplete schooling & 2 & No & 0 & 7 & 12 & depressed / PTSD seperately & 25 & 14 & 14 & 0 & 3+ forms experienced & negative\tabularnewline
37 & incomplete schooling & 1 & Yes & 7 & 9 & 19 & depressed / PTSD seperately & 35 & 13 & 13 & 0 & 2 forms experienced & negative\tabularnewline
35 & incomplete schooling & 2 & Yes & 9 & 6 & 14 & depressed / PTSD seperately & 30 & 9 & 9 & 0 & 3+ forms experienced & negative\tabularnewline
51 & incomplete schooling & 1 & Yes & 12 & 6 & 13 & depressed / PTSD seperately & 48 & 12 & 12 & 1 & 2 forms experienced & positive\tabularnewline
37 & incomplete schooling & 0 & Yes & 12 & 10 & 17 & comorbid ptsd +dep
& 32 & 15 & 15 & 0 & 3+ forms experienced & positive\tabularnewline
22 & matric/post school qualification & 1 & Yes & 0 & 11 & 14 & depressed / PTSD seperately & 19 & 2 & 5 & 0 & 2 forms experienced & negative\tabularnewline
37 & incomplete schooling & 3 & Yes & 0 & 6 & 11 & no MH & 33 & 6 & 9 &
0 & no violence & negative\tabularnewline
35 & incomplete schooling & 4 & No & 6 & 10 & 12 & comorbid ptsd +dep &
30 & 12 & 12 & 0 & no violence & negative\tabularnewline
26 & incomplete schooling & 1 & Yes & 11 & 8 & 19 & no MH & 22 & 12 & 15
& 0 & 2 forms experienced & negative\tabularnewline
26 & incomplete schooling & 2 & Yes & 11 & 6 & 12 & depressed / PTSD seperately & 18 & 5 & 8 & 0 & 2 forms experienced forms experienced form
only & negative\tabularnewline
32 & incomplete schooling & 2 & Yes & 4 & 8 & 11 & depressed / PTSD seperately & 30 & 5 & 8 & 0 & 2 forms experienced & negative\tabularnewline
30 & matric/post school qualification & 1 & No & 9 & 7 & 14 & comorbid
ptsd +dep & 28 & 9 & 9 & 1 & 2 forms experienced & negative\tabularnewline
20 & incomplete schooling & 0 & No & 10 & 8 & 15 & comorbid ptsd +dep &
18 & 11 & 11 & 0 & 2 forms experienced & negative\tabularnewline
23 & incomplete schooling & 1 & Yes & 7 & 9 & 14 & no MH & 24 & 12 & 12
& 0 & 2 forms experienced & negative\tabularnewline
32 & matric/post school qualification & 3 & No & 11 & 10 & 13 & depressed / PTSD seperately & 29 & 11 & 11 & 0 & 2 forms experienced &
positive\tabularnewline
21 & incomplete schooling & 1 & Yes & 11 & 6 & 13 & no MH & 17 & 10 & 10

& 0 & 2 forms experienced & positive\tabularnewline
37 & matric/post school qualification & 0 & No & 10 & 6 & 15 & comorbid
ptsd +dep & 20 & 13 & 16 & 1 & 3+ forms experienced & positive\tabularnewline
35 & incomplete schooling & 1 & No & 13 & 7 & 16 & depressed / PTSD seperately & 17 & 13 & 12 & 1 & 3+ forms experienced & positive\tabularnewline
26 & incomplete schooling & 1 & No & 9 & 6 & 17 & depressed / PTSD seperately & 18 & 12 & 12 & 0 & no violence & negative\tabularnewline
31 & matric/post school qualification & 1 & Yes & 7 & 6 & 13 & comorbid
ptsd +dep & 28 & 11 & 11 & 0 & no violence & negative\tabularnewline
36 & incomplete schooling & 2 & No & 8 & 6 & 15 & depressed / PTSD seperately & 26 & 13 & 13 & 1 & 3+ forms experienced & positive\tabularnewline
22 & matric/post school qualification & 1 & No & 12 & 11 & 21 & depressed / PTSD seperately & 20 & 13 & 13 & 0 & 3+ forms experienced &
negative\tabularnewline
22 & incomplete schooling & 0 & No & 13 & 8 & 15 & comorbid ptsd +dep &
18 & 4 & 7 & 0 & 2 forms experienced forms experienced form only & negative\tabularnewline
43 & matric/post school qualification & 2 & Yes & 10 & 7 & 14 & depressed / PTSD seperately & 37 & 11 & 11 & 0 & no violence & negative\tabularnewline
33 & matric/post school qualification & 1 & No & 0 & 9 & 15 & comorbid
ptsd +dep & 27 & 9 & 8 & 1 & 3+ forms experienced & positive\tabularnewline
25 & incomplete schooling & 1 & Yes & 4 & 8 & 11 & comorbid ptsd +dep &
21 & 11 & 11 & 0 & 2 forms experienced forms experienced form only & negative\tabularnewline
21 & incomplete schooling & 1 & Yes & 13 & 16 & 21 & comorbid ptsd +dep
& 20 & 14 & 14 & 0 & 3+ forms experienced & negative\tabularnewline
46 & matric/post school qualification & 2 & Yes & 10 & 8 & 14 & depressed / PTSD seperately & 35 & 14 & 14 & 0 & 2 forms experienced forms experienced form only & negative\tabularnewline
19 & incomplete schooling & 0 & No & 0 & 6 & 13 & no MH & 18 & 8 & 11 &
0 & 2 forms experienced forms experienced form only & negative\tabularnewline
22 & matric/post school qualification & 1 & Yes & 4 & 6 & 11 & comorbid
ptsd +dep & 21 & 8 & 8 & 0 & 2 forms experienced & negative\tabularnewline
30 & incomplete schooling & 2 & Yes & 11 & 7 & 13 & comorbid ptsd +dep &
27 & 15 & 15 & 0 & 2 forms experienced forms experienced form only & positive\tabularnewline

30 & matric/post school qualification & 2 & No & 5 & 9 & 13 & depressed
/ PTSD seperately & 24 & 12 & 11 & 0 & 3+ forms experienced & negative\tabularnewline
26 & incomplete schooling & 1 & No & 3 & 8 & 11 & depressed / PTSD seperately & 23 & 7 & 7 & 0 & 3+ forms experienced & negative\tabularnewline
41 & incomplete schooling & 3 & Yes & 8 & 14 & 12 & depressed / PTSD seperately & 34 & 3 & 6 & 0 & 3+ forms experienced & positive\tabularnewline
22 & incomplete schooling & 1 & No & 0 & 20 & 19 & no MH & 22 & 7 & 7 &
0 & 3+ forms experienced & negative\tabularnewline
19 & incomplete schooling & 0 & Yes & 11 & 11 & 17 & depressed / PTSD
seperately & 19 & 12 & 12 & 0 & 3+ forms experienced & positive\tabularnewline
24 & matric/post school qualification & 2 & Yes & 8 & 6 & 13 & no MH &
22 & 8 & 8 & 0 & 2 forms experienced forms experienced form only & negative\tabularnewline
27 & incomplete schooling & 2 & No & 12 & 8 & 13 & comorbid ptsd +dep &
18 & 10 & 10 & 0 & 3+ forms experienced & positive\tabularnewline
31 & matric/post school qualification & 11 & Yes & 5 & 8 & 15 & comorbid
ptsd +dep & 29 & 10 & 10 & 0 & 2 forms experienced & negative\tabularnewline
30 & incomplete schooling & 2 & Yes & 0 & 8 & 19 & no MH & 17 & 11 & 11
& 0 & no violence & negative\tabularnewline
34 & incomplete schooling & 0 & No & 13 & 7 & 18 & depressed / PTSD seperately & 27 & 11 & 11 & 1 & 3+ forms experienced & positive\tabularnewline
30 & incomplete schooling & 2 & Yes & 11 & 6 & 14 & comorbid ptsd +dep &
29 & 11 & 11 & 0 & 2 forms experienced & negative\tabularnewline
27 & matric/post school qualification & 1 & Yes & 17 & 10 & 19 & depressed / PTSD seperately & 24 & 5 & 8 & 1 & 3+ forms experienced &
negative\tabularnewline
36 & incomplete schooling & 2 & No & 15 & 6 & 13 & comorbid ptsd +dep &
16 & 11 & 11 & 1 & 2 forms experienced & negative\tabularnewline
26 & matric/post school qualification & 1 & Yes & 15 & 8 & 16 & comorbid
ptsd +dep & 22 & 14 & 14 & 0 & no violence & negative\tabularnewline
25 & matric/post school qualification & 1 & Yes & 10 & 6 & 11 & no MH &
20 & 3 & 6 & 0 & 2 forms experienced forms experienced form only & negative\tabularnewline
33 & incomplete schooling & 1 & No & 6 & 7 & 12 & comorbid ptsd +dep &
30 & 15 & 18 & 1 & 3+ forms experienced & negative\tabularnewline

35 & incomplete schooling & 0 & Yes & 0 & 6 & 14 & comorbid ptsd +dep &
34 & 12 & 12 & 0 & no violence & positive\tabularnewline
29 & incomplete schooling & 4 & Yes & 11 & 7 & 18 & depressed / PTSD seperately & 21 & 8 & 11 & 0 & 2 forms experienced forms experienced form only & positive\tabularnewline
30 & incomplete schooling & 2 & No & 5 & 10 & 15 & depressed / PTSD seperately & 15 & 12 & 12 & 0 & 3+ forms experienced & negative\tabularnewline
23 & incomplete schooling & 2 & Yes & 9 & 6 & 13 & no MH & 22 & 7 & 10 &
0 & 2 forms experienced & negative\tabularnewline
32 & incomplete schooling & 2 & Yes & 12 & 6 & 13 & comorbid ptsd +dep &
19 & 14 & 14 & 0 & 2 forms experienced forms experienced form only & positive\tabularnewline
40 & matric/post school qualification & 5 & Yes & 12 & 13 & 16 & comorbid ptsd +dep & 32 & 13 & 13 & 0 & 2 forms experienced forms experienced form only & positive\tabularnewline
32 & matric/post school qualification & 3 & Yes & 0 & 7 & 11 & comorbid
ptsd +dep & 29 & 10 & 10 & 0 & 2 forms experienced & negative\tabularnewline
24 & matric/post school qualification & 1 & Yes & 13 & 9 & 15 & comorbid
ptsd +dep & 20 & 7 & 4 & 0 & 2 forms experienced & negative\tabularnewline
48 & incomplete schooling & 3 & No & 10 & 6 & 13 & comorbid ptsd +dep &
42 & 16 & 15 & 0 & no violence & negative\tabularnewline
31 & incomplete schooling & 1 & Yes & 0 & 9 & 16 & comorbid ptsd +dep &
28 & 11 & 11 & 0 & 3+ forms experienced & negative\tabularnewline
35 & incomplete schooling & 4 & Yes & 0 & 8 & 12 & comorbid ptsd +dep &
32 & 8 & 8 & 0 & 2 forms experienced & negative\tabularnewline
43 & incomplete schooling & 3 & Yes & 0 & 6 & 21 & depressed / PTSD seperately & 29 & 15 & 15 & 0 & no violence & positive\tabularnewline
38 & incomplete schooling & 2 & Yes & 11 & 18 & 14 & depressed / PTSD
seperately & 35 & 1 & 4 & 0 & 2 forms experienced & positive\tabularnewline
25 & incomplete schooling & 2 & Yes & 10 & 9 & 21 & comorbid ptsd +dep &
23 & 13 & 13 & 0 & 3+ forms experienced & negative\tabularnewline
35 & incomplete schooling & 1 & Yes & 13 & 11 & 17 & no MH & 32 & 6 & 9
& 0 & 3+ forms experienced & positive\tabularnewline
33 & incomplete schooling & 3 & No & 7 & 7 & 13 & depressed / PTSD seperately & 26 & 14 & 17 & 0 & 3+ forms experienced & positive\tabularnewline
27 & incomplete schooling & 1 & No & 6 & 8 & 11 & comorbid ptsd +dep &

23 & 13 & 13 & 0 & 3+ forms experienced & negative\tabularnewline
39 & matric/post school qualification & 1 & Yes & 11 & 7 & 18 & no
MH &
33 & 12 & 15 & 0 & 2 forms experienced & negative\tabularnewline
46 & incomplete schooling & 4 & No & 0 & 7 & 13 & no MH & 33 & 12 &
12 &
0 & 2 forms experienced & positive\tabularnewline
29 & matric/post school qualification & 2 & No & 0 & 9 & 13 &
comorbid
ptsd +dep & 25 & 7 & 7 & 0 & 3+ forms experienced &
negative\tabularnewline
30 & incomplete schooling & 1 & Yes & 11 & 12 & 17 & comorbid ptsd
+dep
& 29 & 15 & 15 & 0 & 2 forms experienced forms experienced form only
&
positive\tabularnewline
40 & incomplete schooling & 2 & No & 12 & 12 & 14 & comorbid ptsd
+dep &
26 & 4 & 7 & 0 & 3+ forms experienced & positive\tabularnewline
28 & incomplete schooling & 2 & No & 11 & 8 & 14 & depressed / PTSD
seperately & 27 & 11 & 14 & 0 & 2 forms experienced forms
experienced
form only & negative\tabularnewline
37 & incomplete schooling & 3 & No & 12 & 9 & 14 & depressed / PTSD
seperately & 35 & 15 & 15 & 0 & no violence &
positive\tabularnewline
22 & incomplete schooling & 0 & No & 10 & 8 & 19 & depressed / PTSD
seperately & 17 & 12 & 12 & 0 & 2 forms experienced &
negative\tabularnewline
24 & incomplete schooling & 1 & Yes & 9 & 7 & 15 & depressed / PTSD
seperately & 24 & 10 & 10 & 0 & 2 forms experienced forms
experienced
form only & negative\tabularnewline
35 & incomplete schooling & 2 & No & 11 & 8 & 19 & depressed / PTSD
seperately & 30 & 8 & 11 & 0 & 2 forms experienced forms experienced
form only & negative\tabularnewline
41 & incomplete schooling & 3 & Yes & 10 & 8 & 15 & depressed / PTSD
seperately & 36 & 11 & 11 & 0 & 3+ forms experienced &
negative\tabularnewline
41 & incomplete schooling & 3 & Yes & 0 & 6 & 21 & no MH & 23 & 15 &
15
& 0 & 2 forms experienced & positive\tabularnewline
21 & incomplete schooling & 0 & Yes & 12 & 9 & 21 & comorbid ptsd
+dep &
20 & 8 & 11 & 1 & 2 forms experienced & negative\tabularnewline
23 & incomplete schooling & 0 & Yes & 17 & 9 & 21 & depressed / PTSD
seperately & 19 & 10 & 13 & 0 & no violence &
negative\tabularnewline
25 & incomplete schooling & 1 & Yes & 14 & 8 & 13 & comorbid ptsd
+dep &
23 & 9 & 9 & 0 & 2 forms experienced & negative\tabularnewline
28 & incomplete schooling & 3 & Yes & 10 & 8 & 15 & depressed / PTSD
seperately & 24 & 8 & 11 & 1 & 2 forms experienced &
negative\tabularnewline

39 & matric/post school qualification & 2 & Yes & 13 & 6 & 12 & no MH &
31 & 2 & 5 & 0 & 2 forms experienced & negative\tabularnewline
30 & matric/post school qualification & 3 & Yes & 4 & 10 & 16 & comorbid
ptsd +dep & 25 & 8 & 8 & 0 & 2 forms experienced & negative\tabularnewline
19 & incomplete schooling & 1 & Yes & 10 & 7 & 18 & depressed / PTSD seperately & 17 & 7 & 10 & 0 & 2 forms experienced & negative\tabularnewline
39 & incomplete schooling & 2 & Yes & 10 & 6 & 9 & no MH & 27 & 6 & 9 &
1 & 3+ forms experienced & negative\tabularnewline
33 & incomplete schooling & 3 & No & 0 & 7 & 13 & depressed / PTSD seperately & 30 & 14 & 14 & 0 & 3+ forms experienced & negative\tabularnewline
36 & incomplete schooling & 2 & Yes & 11 & 7 & 17 & comorbid ptsd +dep &
33 & 11 & 11 & 0 & no violence & positive\tabularnewline
34 & incomplete schooling & 1 & No & 8 & 8 & 15 & comorbid ptsd +dep &
31 & 8 & 8 & 0 & 2 forms experienced & negative\tabularnewline
23 & matric/post school qualification & 1 & No & 13 & 6 & 13 & no MH &
22 & 4 & 7 & 0 & no violence & negative\tabularnewline
24 & incomplete schooling & 1 & Yes & 11 & 6 & 15 & comorbid ptsd +dep &
19 & 13 & 13 & 0 & 3+ forms experienced & negative\tabularnewline
19 & matric/post school qualification & 0 & No & 10 & 6 & 13 & comorbid
ptsd +dep & 18 & 11 & 11 & 0 & 2 forms experienced forms experienced form only & negative\tabularnewline
30 & matric/post school qualification & 1 & Yes & 14 & 9 & 16 & no MH &
23 & 10 & 13 & 0 & 3+ forms experienced & negative\tabularnewline
39 & incomplete schooling & 3 & Yes & 0 & 9 & 10 & comorbid ptsd +dep &
21 & 7 & 10 & 1 & 3+ forms experienced & positive\tabularnewline
36 & incomplete schooling & 2 & No & 10 & 8 & 13 & comorbid ptsd +dep &
32 & 10 & 13 & 1 & no violence & positive\tabularnewline
41 & incomplete schooling & 3 & Yes & 9 & 7 & 12 & comorbid ptsd +dep &
14 & 7 & 10 & 1 & 2 forms experienced & negative\tabularnewline
40 & matric/post school qualification & 2 & Yes & 6 & 7 & 12 & depressed
/ PTSD seperately & 34 & 3 & 6 & 0 & 3+ forms experienced & negative\tabularnewline
31 & matric/post school qualification & 1 & Yes & 11 & 8 & 14 & no MH &
29 & 11 & 11 & 0 & 2 forms experienced forms experienced form only & negative\tabularnewline
37 & incomplete schooling & 1 & Yes & 13 & 7 & 17 & no MH & 34 & 11 & 11

& 0 & 2 forms experienced forms experienced form only &
positive\tabularnewline
37 & incomplete schooling & 4 & Yes & 12 & 11 & 16 & depressed /
PTSD
seperately & 32 & 15 & 15 & 0 & 2 forms experienced &
positive\tabularnewline
40 & incomplete schooling & 4 & Yes & 11 & 10 & 15 & no MH & 38 & 11
&
11 & 0 & no violence & positive\tabularnewline
36 & incomplete schooling & 5 & Yes & 6 & 9 & 12 & comorbid ptsd
+dep &
28 & 11 & 14 & 0 & 3+ forms experienced & positive\tabularnewline
24 & incomplete schooling & 0 & Yes & 15 & 10 & 20 & depressed /
PTSD
seperately & 23 & 11 & 11 & 1 & 2 forms experienced &
positive\tabularnewline
22 & incomplete schooling & 0 & Yes & 0 & 7 & 15 & comorbid ptsd
+dep &
19 & 5 & 8 & 0 & 3+ forms experienced & negative\tabularnewline
22 & incomplete schooling & 2 & Yes & 0 & 7 & 14 & comorbid ptsd
+dep &
21 & 10 & 10 & 0 & 2 forms experienced & positive\tabularnewline
36 & incomplete schooling & 2 & Yes & 11 & 9 & 13 & comorbid ptsd
+dep &
33 & 11 & 11 & 0 & no violence & positive\tabularnewline
46 & incomplete schooling & 3 & No & 0 & 9 & 19 & depressed / PTSD
seperately & 44 & 7 & 10 & 1 & 2 forms experienced forms experienced
form only & positive\tabularnewline
21 & matric/post school qualification & 0 & Yes & 4 & 6 & 14 & no MH
&
18 & 4 & 7 & 0 & 2 forms experienced & negative\tabularnewline
43 & incomplete schooling & 4 & Yes & 12 & 8 & 17 & depressed / PTSD
seperately & 36 & 13 & 13 & 0 & 2 forms experienced &
positive\tabularnewline
18 & incomplete schooling & 0 & Yes & 6 & 8 & 14 & depressed / PTSD
seperately & 18 & 1 & 4 & 0 & 2 forms experienced forms experienced
form
only & negative\tabularnewline
26 & incomplete schooling & 2 & No & 6 & 10 & 11 & comorbid ptsd
+dep &
16 & 14 & 17 & 1 & 3+ forms experienced & positive\tabularnewline
40 & incomplete schooling & 4 & Yes & 14 & 6 & 20 & depressed / PTSD
seperately & 34 & 9 & 12 & 0 & 3+ forms experienced &
positive\tabularnewline
27 & matric/post school qualification & 2 & Yes & 10 & 8 & 12 &
depressed / PTSD seperately & 22 & 18 & 18 & 0 & 2 forms experienced
forms experienced form only & negative\tabularnewline
31 & incomplete schooling & 2 & Yes & 13 & 8 & 14 & depressed / PTSD
seperately & 28 & 15 & 15 & 1 & 2 forms experienced &
positive\tabularnewline
31 & matric/post school qualification & 0 & No & 13 & 11 & 13 &
comorbid
ptsd +dep & 28 & 1 & 4 & 1 & 3+ forms experienced &
positive\tabularnewline

43 & matric/post school qualification & 3 & Yes & 11 & 6 & 13 &
depressed / PTSD seperately & 39 & 9 & 12 & 0 & 2 forms experienced
forms experienced form only & negative\tabularnewline
22 & incomplete schooling & 0 & No & 10 & 6 & 13 & depressed / PTSD
seperately & 20 & 12 & 12 & 0 & 2 forms experienced forms
experienced
form only & negative\tabularnewline
40 & matric/post school qualification & 2 & Yes & 5 & 9 & 12 &
depressed
/ PTSD seperately & 37 & 12 & 12 & 1 & 3+ forms experienced &
negative\tabularnewline
31 & incomplete schooling & 2 & No & 4 & 6 & 14 & comorbid ptsd +dep
&
31 & 11 & 11 & 1 & 3+ forms experienced & positive\tabularnewline
35 & incomplete schooling & 3 & Yes & 16 & 8 & 20 & depressed / PTSD
seperately & 27 & 17 & 17 & 1 & 3+ forms experienced &
negative\tabularnewline
25 & incomplete schooling & 3 & Yes & 6 & 7 & 12 & comorbid ptsd
+dep &
15 & 13 & 13 & 0 & 3+ forms experienced & negative\tabularnewline
25 & incomplete schooling & 0 & Yes & 6 & 6 & 11 & no MH & 20 & 8 &
8 &
0 & 2 forms experienced forms experienced form only &
negative\tabularnewline
27 & incomplete schooling & 2 & Yes & 10 & 7 & 13 & comorbid ptsd
+dep &
24 & 12 & 12 & 1 & 2 forms experienced forms experienced form only &
negative\tabularnewline
28 & incomplete schooling & 3 & Yes & 14 & 6 & 13 & no MH & 22 & 12
& 12
& 0 & 2 forms experienced forms experienced form only &
positive\tabularnewline
29 & matric/post school qualification & 1 & Yes & 14 & 10 & 14 & no
MH &
25 & 4 & 7 & 0 & 3+ forms experienced & positive\tabularnewline
32 & incomplete schooling & 2 & Yes & 8 & 6 & 18 & depressed / PTSD
seperately & 18 & 11 & 11 & 1 & no violence &
positive\tabularnewline
30 & matric/post school qualification & 0 & Yes & 14 & 11 & 17 &
depressed / PTSD seperately & 24 & 13 & 13 & 1 & 3+ forms
experienced &
negative\tabularnewline
22 & incomplete schooling & 1 & Yes & 10 & 6 & 13 & no MH & 22 & 12
& 12
& 0 & 2 forms experienced forms experienced form only &
negative\tabularnewline
22 & matric/post school qualification & 1 & Yes & 12 & 6 & 14 & no
MH &
21 & 11 & 11 & 0 & no violence & negative\tabularnewline
18 & incomplete schooling & 0 & No & 0 & 11 & 17 & comorbid ptsd
+dep &
16 & 13 & 12 & 0 & 3+ forms experienced & negative\tabularnewline
34 & incomplete schooling & 2 & No & 5 & 8 & 11 & depressed / PTSD
seperately & 18 & 8 & 11 & 0 & 2 forms experienced forms experienced

form only & positive\tabularnewline
20 & matric/post school qualification & 0 & Yes & 7 & 8 & 15 & depressed
/ PTSD seperately & 20 & 15 & 15 & 0 & 2 forms experienced & negative\tabularnewline
29 & matric/post school qualification & 0 & Yes & 12 & 6 & 15 & no MH &
19 & 11 & 11 & 0 & 3+ forms experienced & negative\tabularnewline
21 & incomplete schooling & 2 & Yes & 10 & 8 & 14 & comorbid ptsd +dep &
19 & 9 & 9 & 0 & 2 forms experienced & positive\tabularnewline
30 & incomplete schooling & 1 & No & 11 & 6 & 13 & comorbid ptsd +dep &
27 & 9 & 12 & 0 & no violence & positive\tabularnewline
22 & matric/post school qualification & 0 & Yes & 3 & 8 & 13 & comorbid
ptsd +dep & 18 & 9 & 9 & 0 & 3+ forms experienced & negative\tabularnewline
30 & incomplete schooling & 1 & Yes & 10 & 11 & 14 & no MH & 25 & 8 & 8
& 0 & 3+ forms experienced & negative\tabularnewline
40 & incomplete schooling & 5 & No & 11 & 8 & 13 & depressed / PTSD
seperately & 35 & 3 & 6 & 0 & 3+ forms experienced & positive\tabularnewline
42 & matric/post school qualification & 2 & Yes & 12 & 8 & 14 & depressed / PTSD seperately & 40 & 11 & 11 & 0 & no violence & positive\tabularnewline
23 & matric/post school qualification & 2 & Yes & 10 & 7 & 13 & comorbid
ptsd +dep & 22 & 11 & 11 & 0 & 2 forms experienced forms experienced
form only & negative\tabularnewline
33 & incomplete schooling & 0 & Yes & 0 & 8 & 13 & no MH & 26 & 3 & 6 &
0 & 3+ forms experienced & negative\tabularnewline
18 & incomplete schooling & 1 & No & 11 & 10 & 14 & comorbid ptsd +dep &
17 & 16 & 16 & 0 & 2 forms experienced forms experienced form only & negative\tabularnewline
29 & matric/post school qualification & 3 & No & 0 & 6 & 17 & depressed
/ PTSD seperately & 22 & 6 & 9 & 0 & no violence & negative\tabularnewline
24 & incomplete schooling & 1 & No & 0 & 6 & 13 & depressed / PTSD
seperately & 24 & 6 & 9 & 0 & 2 forms experienced & negative\tabularnewline
41 & incomplete schooling & 3 & Yes & 9 & 7 & 14 & comorbid ptsd +dep &
25 & 9 & 9 & 0 & 3+ forms experienced & negative\tabularnewline
18 & incomplete schooling & 1 & Yes & 10 & 6 & 18 & depressed / PTSD
seperately & 16 & 14 & 17 & 0 & 3+ forms experienced & negative\tabularnewline
28 & incomplete schooling & 0 & Yes & 14 & 12 & 16 & depressed / PTSD
seperately & 29 & 13 & 13 & 1 & 3+ forms experienced &

positive\tabularnewline
25 & incomplete schooling & 3 & Yes & 11 & 6 & 13 & comorbid ptsd +dep &
24 & 9 & 12 & 0 & no violence & negative\tabularnewline
36 & incomplete schooling & 2 & Yes & 0 & 6 & 13 & no MH & 31 & 2 & 5 &
1 & 2 forms experienced & positive\tabularnewline
33 & incomplete schooling & 3 & No & 10 & 6 & 12 & no MH & 29 & 8 & 8 &
0 & no violence & positive\tabularnewline
41 & incomplete schooling & 3 & Yes & 5 & 6 & 14 & depressed / PTSD seperately & 38 & 11 & 11 & 0 & 3+ forms experienced & negative\tabularnewline
34 & incomplete schooling & 5 & Yes & 7 & 6 & 11 & depressed / PTSD seperately & 25 & 10 & 10 & 1 & no violence & positive\tabularnewline
32 & incomplete schooling & 4 & Yes & 4 & 8 & 13 & depressed / PTSD seperately & 30 & 7 & 10 & 0 & 2 forms experienced forms experienced form only & positive\tabularnewline
39 & matric/post school qualification & 0 & Yes & 4 & 9 & 11 & depressed
/ PTSD seperately & 27 & 8 & 8 & 1 & 3+ forms experienced & positive\tabularnewline
31 & matric/post school qualification & 3 & Yes & 11 & 6 & 13 & no MH &
30 & 10 & 10 & 0 & 2 forms experienced & negative\tabularnewline
34 & incomplete schooling & 2 & No & 0 & 9 & 12 & comorbid ptsd +dep &
31 & 6 & 5 & 0 & 2 forms experienced & positive\tabularnewline
20 & incomplete schooling & 1 & Yes & 10 & 9 & 16 & depressed / PTSD seperately & 20 & 15 & 15 & 0 & 3+ forms experienced & negative\tabularnewline
25 & matric/post school qualification & 1 & Yes & 14 & 6 & 11 & no MH &
23 & 13 & 16 & 0 & 2 forms experienced & positive\tabularnewline
27 & matric/post school qualification & 2 & No & 9 & 6 & 12 & no MH & 24
& 15 & 18 & 0 & 2 forms experienced forms experienced form only & positive\tabularnewline
22 & incomplete schooling & 1 & No & 10 & 6 & 13 & no MH & 22 & 6 & 9 &
0 & no violence & negative\tabularnewline
21 & incomplete schooling & 1 & Yes & 0 & 8 & 12 & comorbid ptsd +dep &
18 & 8 & 8 & 0 & 3+ forms experienced & negative\tabularnewline
47 & incomplete schooling & 0 & No & 11 & 9 & 13 & depressed / PTSD seperately & 37 & 5 & 8 & 0 & 2 forms experienced & negative\tabularnewline
31 & matric/post school qualification & 3 & No & 10 & 6 & 13 & depressed
/ PTSD seperately & 28 & 12 & 12 & 0 & 2 forms experienced forms experienced form only & positive\tabularnewline
30 & incomplete schooling & 3 & Yes & 10 & 9 & 18 & no MH & 18 & 5 & 8 &

1 & 3+ forms experienced & negative\tabularnewline
33 & incomplete schooling & 1 & Yes & 7 & 16 & 21 & comorbid ptsd
+dep &
22 & 11 & 11 & 0 & 3+ forms experienced & positive\tabularnewline
19 & incomplete schooling & 0 & Yes & 3 & 7 & 11 & comorbid ptsd
+dep &
17 & 8 & 8 & 0 & 3+ forms experienced & negative\tabularnewline
23 & incomplete schooling & 2 & Yes & 9 & 10 & 12 & comorbid ptsd
+dep &
16 & 14 & 14 & 0 & 3+ forms experienced & negative\tabularnewline
28 & matric/post school qualification & 1 & Yes & 12 & 6 & 13 &
comorbid
ptsd +dep & 26 & 11 & 11 & 0 & 2 forms experienced forms experienced
form only & negative\tabularnewline
32 & matric/post school qualification & 5 & Yes & 10 & 9 & 22 &
depressed / PTSD seperately & 25 & 11 & 11 & 0 & 3+ forms
experienced &
positive\tabularnewline
31 & incomplete schooling & 1 & Yes & 6 & 8 & 13 & comorbid ptsd
+dep &
19 & 13 & 13 & 0 & 3+ forms experienced & negative\tabularnewline
28 & incomplete schooling & 0 & Yes & 10 & 7 & 13 & depressed / PTSD
seperately & 27 & 11 & 11 & 0 & 2 forms experienced forms
experienced
form only & positive\tabularnewline
20 & incomplete schooling & 1 & Yes & 0 & 8 & 23 & comorbid ptsd
+dep &
17 & 6 & 9 & 0 & 2 forms experienced forms experienced form only &
negative\tabularnewline
24 & incomplete schooling & 1 & Yes & 12 & 9 & 19 & no MH & 23 & 6 &
9 &
0 & no violence & negative\tabularnewline
35 & incomplete schooling & 3 & Yes & 7 & 6 & 13 & depressed / PTSD
seperately & 27 & 12 & 15 & 0 & 2 forms experienced &
positive\tabularnewline
23 & matric/post school qualification & 1 & No & 11 & 8 & 13 &
comorbid
ptsd +dep & 21 & 9 & 12 & 0 & 2 forms experienced forms experienced
form
only & negative\tabularnewline
28 & incomplete schooling & 0 & Yes & 11 & 8 & 16 & comorbid ptsd
+dep &
21 & 7 & 7 & 0 & 3+ forms experienced & negative\tabularnewline
26 & incomplete schooling & 2 & Yes & 8 & 9 & 19 & depressed / PTSD
seperately & 24 & 6 & 9 & 0 & 2 forms experienced &
positive\tabularnewline
28 & incomplete schooling & 2 & Yes & 10 & 10 & 14 & comorbid ptsd
+dep
& 18 & 15 & 15 & 0 & 2 forms experienced & positive\tabularnewline
22 & incomplete schooling & 1 & Yes & 13 & 7 & 13 & depressed / PTSD
seperately & 20 & 12 & 12 & 0 & 2 forms experienced &
positive\tabularnewline
46 & incomplete schooling & 2 & Yes & 6 & 8 & 12 & comorbid ptsd
+dep &

40 & 11 & 11 & 0 & 3+ forms experienced & negative\tabularnewline
26 & matric/post school qualification & 0 & Yes & 0 & 9 & 15 & no MH &
22 & 9 & 8 & 0 & no violence & negative\tabularnewline
29 & incomplete schooling & 0 & No & 10 & 9 & 11 & no MH & 26 & 9 & 12 &
0 & 2 forms experienced forms experienced form only & negative\tabularnewline
37 & incomplete schooling & 3 & Yes & 11 & 7 & 16 & depressed / PTSD seperately & 37 & 11 & 11 & 1 & 2 forms experienced & positive\tabularnewline
23 & matric/post school qualification & 0 & Yes & 5 & 7 & 13 & depressed
/ PTSD seperately & 21 & 12 & 12 & 0 & 2 forms experienced & negative\tabularnewline
29 & incomplete schooling & 3 & Yes & 7 & 6 & 18 & depressed / PTSD seperately & 22 & 10 & 10 & 0 & 3+ forms experienced & negative\tabularnewline
42 & incomplete schooling & 1 & Yes & 4 & 9 & 16 & comorbid ptsd +dep &
25 & 10 & 10 & 0 & 3+ forms experienced & positive\tabularnewline
34 & incomplete schooling & 2 & Yes & 0 & 6 & 13 & depressed / PTSD seperately & 33 & 8 & 11 & 0 & 3+ forms experienced & positive\tabularnewline
38 & incomplete schooling & 0 & Yes & 4 & 10 & 14 & depressed / PTSD seperately & 17 & 7 & 6 & 0 & 3+ forms experienced & positive\tabularnewline
28 & incomplete schooling & 0 & Yes & 13 & 8 & 23 & depressed / PTSD seperately & 24 & 4 & 7 & 1 & 2 forms experienced forms experienced form
only & positive\tabularnewline
40 & incomplete schooling & 0 & No & 8 & 8 & 17 & comorbid ptsd +dep &
38 & 14 & 17 & 0 & 2 forms experienced & positive\tabularnewline
32 & matric/post school qualification & 2 & No & 14 & 8 & 16 & comorbid
ptsd +dep & 28 & 17 & 16 & 0 & 2 forms experienced forms experienced form only & positive\tabularnewline
20 & incomplete schooling & 0 & No & 15 & 7 & 13 & no MH & 19 & 12 & 12
& 0 & 2 forms experienced forms experienced form only & negative\tabularnewline
22 & incomplete schooling & 1 & No & 0 & 8 & 13 & no MH & 17 & 13 & 13 &
0 & 2 forms experienced & positive\tabularnewline
38 & incomplete schooling & 2 & No & 11 & 6 & 16 & comorbid ptsd +dep &
33 & 16 & 16 & 1 & 2 forms experienced forms experienced form only & positive\tabularnewline
43 & incomplete schooling & 2 & Yes & 8 & 6 & 18 & depressed / PTSD seperately & 35 & 14 & 14 & 0 & 2 forms experienced forms experienced
form only & negative\tabularnewline
18 & incomplete schooling & 0 & Yes & 9 & 6 & 21 & depressed / PTSD

seperately & 16 & 15 & 15 & 0 & no violence &
negative\tabularnewline
19 & incomplete schooling & 1 & Yes & 12 & 8 & 15 & comorbid ptsd
+dep &
18 & 15 & 15 & 0 & no violence & negative\tabularnewline
38 & incomplete schooling & 2 & Yes & 0 & 11 & 18 & comorbid ptsd
+dep &
20 & 10 & 10 & 1 & 3+ forms experienced & positive\tabularnewline
26 & matric/post school qualification & 0 & No & 5 & 8 & 18 &
comorbid
ptsd +dep & 24 & 2 & 5 & 0 & 2 forms experienced forms experienced
form
only & negative\tabularnewline
51 & incomplete schooling & 0 & Yes & 10 & 10 & 17 & depressed /
PTSD
seperately & 32 & 11 & 11 & 1 & 3+ forms experienced &
positive\tabularnewline
29 & incomplete schooling & 1 & Yes & 10 & 6 & 13 & depressed / PTSD
seperately & 24 & 6 & 9 & 0 & 2 forms experienced &
positive\tabularnewline
25 & incomplete schooling & 1 & Yes & 11 & 6 & 20 & no MH & 22 & 7 &
10
& 0 & 2 forms experienced forms experienced form only &
positive\tabularnewline
27 & incomplete schooling & 2 & Yes & 11 & 8 & 17 & no MH & 24 & 2 &
5 &
0 & 3+ forms experienced & positive\tabularnewline
33 & incomplete schooling & 2 & No & 8 & 12 & 15 & depressed / PTSD
seperately & 27 & 11 & 14 & 0 & 3+ forms experienced &
positive\tabularnewline
20 & incomplete schooling & 1 & Yes & 10 & 8 & 18 & no MH & 19 & 14
& 14
& 0 & no violence & negative\tabularnewline
48 & incomplete schooling & 3 & Yes & 0 & 8 & 11 & comorbid ptsd
+dep &
34 & 13 & 13 & 0 & 3+ forms experienced & negative\tabularnewline
34 & incomplete schooling & 4 & Yes & 11 & 8 & 15 & depressed / PTSD
seperately & 28 & 7 & 6 & 0 & no violence & positive\tabularnewline
35 & incomplete schooling & 2 & No & 7 & 7 & 19 & comorbid ptsd +dep
&
22 & 10 & 13 & 0 & 3+ forms experienced & positive\tabularnewline
34 & matric/post school qualification & 2 & Yes & 8 & 6 & 13 & no MH
&
32 & 7 & 10 & 1 & 2 forms experienced & negative\tabularnewline
34 & incomplete schooling & 1 & Yes & 15 & 7 & 17 & depressed / PTSD
seperately & 30 & 4 & 7 & 0 & 2 forms experienced forms experienced
form
only & negative\tabularnewline
31 & incomplete schooling & 3 & Yes & 14 & 14 & 13 & no MH & 18 & 8
& 8
& 0 & 3+ forms experienced & positive\tabularnewline
35 & incomplete schooling & 1 & Yes & 8 & 6 & 14 & depressed / PTSD
seperately & 34 & 14 & 14 & 0 & 2 forms experienced &
negative\tabularnewline

32 & matric/post school qualification & 3 & Yes & 11 & 6 & 17 &
depressed / PTSD seperately & 31 & 16 & 16 & 0 & 3+ forms
experienced &
negative\tabularnewline
23 & incomplete schooling & 0 & No & 11 & 6 & 14 & depressed / PTSD
seperately & 18 & 14 & 14 & 0 & 2 forms experienced forms
experienced
form only & negative\tabularnewline
30 & matric/post school qualification & 1 & Yes & 10 & 6 & 13 &
depressed / PTSD seperately & 28 & 11 & 11 & 0 & no violence &
positive\tabularnewline
25 & incomplete schooling & 1 & Yes & 9 & 8 & 17 & depressed / PTSD
seperately & 23 & 8 & 11 & 0 & 2 forms experienced &
positive\tabularnewline
30 & matric/post school qualification & 1 & Yes & 0 & 6 & 17 & no MH
&
27 & 10 & 13 & 0 & 3+ forms experienced & positive\tabularnewline
48 & incomplete schooling & 0 & No & 10 & 10 & 16 & comorbid ptsd
+dep &
28 & 1 & 4 & 0 & 2 forms experienced & positive\tabularnewline
28 & incomplete schooling & 3 & Yes & 11 & 6 & 17 & depressed / PTSD
seperately & 25 & 4 & 4 & 0 & 2 forms experienced &
negative\tabularnewline
42 & incomplete schooling & 3 & Yes & 12 & 6 & 11 & no MH & 39 & 4 &
7 &
0 & 3+ forms experienced & negative\tabularnewline
30 & incomplete schooling & 0 & Yes & 12 & 12 & 15 & comorbid ptsd
+dep
& 28 & 13 & 12 & 1 & 2 forms experienced & positive\tabularnewline
27 & incomplete schooling & 2 & Yes & 10 & 9 & 16 & comorbid ptsd
+dep &
21 & 13 & 16 & 0 & 3+ forms experienced & positive\tabularnewline
22 & incomplete schooling & 2 & Yes & 5 & 6 & 20 & no MH & 19 & 8 &
8 &
0 & 2 forms experienced forms experienced form only &
positive\tabularnewline
31 & incomplete schooling & 2 & Yes & 10 & 7 & 13 & depressed / PTSD
seperately & 24 & 9 & 9 & 0 & no violence & negative\tabularnewline
26 & matric/post school qualification & 1 & Yes & 11 & 6 & 13 &
depressed / PTSD seperately & 23 & 12 & 12 & 0 & 2 forms experienced
forms experienced form only & negative\tabularnewline
45 & incomplete schooling & 2 & Yes & 11 & 17 & 20 & comorbid ptsd
+dep
& 18 & 13 & 13 & 1 & 3+ forms experienced & positive\tabularnewline
41 & incomplete schooling & 2 & Yes & 14 & 8 & 14 & no MH & 41 & 15
& 15
& 1 & 2 forms experienced & positive\tabularnewline
20 & matric/post school qualification & 2 & No & 0 & 9 & 10 &
comorbid
ptsd +dep & 22 & 0 & 3 & 0 & 3+ forms experienced &
negative\tabularnewline
43 & incomplete schooling & 2 & No & 6 & 9 & 12 & comorbid ptsd +dep
&
34 & 9 & 8 & 0 & 3+ forms experienced & negative\tabularnewline

25 & incomplete schooling & 1 & No & 9 & 8 & 18 & depressed / PTSD seperately & 17 & 6 & 9 & 1 & 2 forms experienced forms experienced form
only & negative\tabularnewline
27 & incomplete schooling & 0 & Yes & 6 & 9 & 12 & comorbid ptsd +dep &
21 & 12 & 12 & 1 & 2 forms experienced & positive\tabularnewline
47 & incomplete schooling & 3 & No & 12 & 8 & 11 & comorbid ptsd +dep &
30 & 7 & 10 & 0 & 3+ forms experienced & positive\tabularnewline
26 & matric/post school qualification & 1 & Yes & 11 & 8 & 13 & depressed / PTSD seperately & 19 & 11 & 11 & 0 & 2 forms experienced forms experienced form only & negative\tabularnewline
28 & matric/post school qualification & 0 & Yes & 9 & 8 & 13 & comorbid
ptsd +dep & 24 & 12 & 11 & 0 & 3+ forms experienced & negative\tabularnewline
30 & incomplete schooling & 1 & Yes & 0 & 10 & 17 & depressed / PTSD seperately & 27 & 15 & 15 & 0 & 3+ forms experienced & positive\tabularnewline
35 & matric/post school qualification & 4 & Yes & 0 & 9 & 15 & depressed
/ PTSD seperately & 33 & 14 & 14 & 0 & 2 forms experienced & positive\tabularnewline
43 & incomplete schooling & 2 & Yes & 13 & 6 & 13 & depressed / PTSD seperately & 41 & 11 & 11 & 0 & 2 forms experienced forms experienced
form only & negative\tabularnewline
27 & matric/post school qualification & 1 & Yes & 4 & 7 & 16 & comorbid
ptsd +dep & 15 & 4 & 7 & 0 & 2 forms experienced & negative\tabularnewline
28 & incomplete schooling & 2 & Yes & 4 & 8 & 12 & depressed / PTSD seperately & 21 & 11 & 11 & 1 & 3+ forms experienced & positive\tabularnewline
40 & matric/post school qualification & 6 & Yes & 11 & 8 & 11 & comorbid
ptsd +dep & 19 & 8 & 11 & 0 & 3+ forms experienced & negative\tabularnewline
43 & incomplete schooling & 1 & No & 9 & 8 & 19 & comorbid ptsd +dep &
18 & 14 & 14 & 0 & 3+ forms experienced & positive\tabularnewline
21 & incomplete schooling & 2 & Yes & 14 & 6 & 13 & depressed / PTSD seperately & 19 & 9 & 9 & 0 & 2 forms experienced forms experienced form
only & negative\tabularnewline
25 & incomplete schooling & 2 & Yes & 13 & 6 & 16 & depressed / PTSD seperately & 23 & 11 & 14 & 0 & 2 forms experienced & positive\tabularnewline
25 & incomplete schooling & 2 & No & 13 & 6 & 11 & no MH & 24 & 8 & 8 &
0 & no violence & negative\tabularnewline
34 & matric/post school qualification & 3 & No & 4 & 6 & 11 & no MH & 32

& 8 & 8 & 0 & 2 forms experienced & negative\tabularnewline
26 & matric/post school qualification & 1 & Yes & 15 & 9 & 11 & no MH &
18 & 6 & 9 & 0 & 2 forms experienced & negative\tabularnewline
39 & incomplete schooling & 2 & Yes & 0 & 6 & 11 & depressed / PTSD seperately & 38 & 5 & 4 & 0 & 2 forms experienced & negative\tabularnewline
29 & incomplete schooling & 1 & No & 0 & 6 & 11 & no MH & 20 & 6 & 9 & 0
& 2 forms experienced & positive\tabularnewline
33 & incomplete schooling & 2 & Yes & 11 & 6 & 18 & comorbid ptsd +dep &
29 & 11 & 11 & 0 & 2 forms experienced forms experienced form only & negative\tabularnewline
35 & matric/post school qualification & 2 & No & 3 & 9 & 15 & depressed
/ PTSD seperately & 30 & 5 & 8 & 0 & 2 forms experienced & negative\tabularnewline
25 & incomplete schooling & 0 & Yes & 0 & 9 & 13 & comorbid ptsd +dep &
22 & 8 & 8 & 0 & 2 forms experienced & positive\tabularnewline
24 & incomplete schooling & 0 & No & 5 & 14 & 18 & depressed / PTSD seperately & 17 & 3 & 6 & 1 & 2 forms experienced & negative\tabularnewline
33 & matric/post school qualification & 2 & Yes & 5 & 8 & 14 & depressed
/ PTSD seperately & 30 & 8 & 8 & 0 & 3+ forms experienced & negative\tabularnewline
28 & incomplete schooling & 1 & Yes & 13 & 6 & 13 & comorbid ptsd +dep &
26 & 3 & 6 & 1 & 2 forms experienced & negative\tabularnewline
19 & incomplete schooling & 0 & No & 0 & 7 & 19 & no MH & 17 & 10 & 10 &
0 & 3+ forms experienced & negative\tabularnewline
26 & incomplete schooling & 2 & Yes & 12 & 6 & 15 & comorbid ptsd +dep &
22 & 7 & 10 & 0 & 2 forms experienced & positive\tabularnewline
18 & incomplete schooling & 1 & No & 0 & 6 & 11 & depressed / PTSD seperately & 17 & 2 & 5 & 0 & no violence & negative\tabularnewline
43 & incomplete schooling & 2 & No & 16 & 8 & 15 & depressed / PTSD seperately & 42 & 16 & 16 & 0 & 2 forms experienced & negative\tabularnewline
26 & incomplete schooling & 2 & Yes & 6 & 7 & 13 & comorbid ptsd +dep &
24 & 7 & 7 & 0 & 3+ forms experienced & negative\tabularnewline
26 & incomplete schooling & 2 & Yes & 14 & 12 & 18 & no MH & 24 & 3 & 6
& 0 & 3+ forms experienced & negative\tabularnewline
22 & matric/post school qualification & 0 & Yes & 7 & 9 & 19 & depressed
/ PTSD seperately & 20 & 9 & 9 & 0 & no violence & negative\tabularnewline
43 & incomplete schooling & 0 & No & 15 & 8 & 14 & comorbid ptsd +dep &

16 & 11 & 11 & 0 & 2 forms experienced forms experienced form only & positive\tabularnewline
38 & incomplete schooling & 2 & Yes & 10 & 6 & 12 & no MH & 26 & 3 & 6 &
0 & 2 forms experienced & positive\tabularnewline
47 & incomplete schooling & 1 & No & 12 & 12 & 18 & comorbid ptsd +dep &
26 & 15 & 15 & 0 & 3+ forms experienced & positive\tabularnewline
18 & incomplete schooling & 0 & Yes & 9 & 7 & 18 & depressed / PTSD seperately & 16 & 5 & 8 & 0 & no violence & negative\tabularnewline
24 & incomplete schooling & 1 & No & 11 & 6 & 14 & comorbid ptsd +dep &
20 & 12 & 12 & 0 & 2 forms experienced forms experienced form only & negative\tabularnewline
40 & incomplete schooling & 4 & Yes & 13 & 9 & 12 & depressed / PTSD seperately & 30 & 8 & 8 & 0 & 2 forms experienced & positive\tabularnewline
42 & incomplete schooling & 2 & Yes & 0 & 9 & 12 & comorbid ptsd +dep &
37 & 9 & 9 & 0 & 3+ forms experienced & negative\tabularnewline
28 & matric/post school qualification & 1 & Yes & 0 & 8 & 13 & comorbid
ptsd +dep & 27 & 11 & 10 & 0 & 2 forms experienced forms experienced form only & negative\tabularnewline
29 & incomplete schooling & 2 & Yes & 10 & 7 & 13 & depressed / PTSD seperately & 27 & 4 & 4 & 0 & 2 forms experienced & negative\tabularnewline
34 & incomplete schooling & 4 & No & 14 & 6 & 13 & comorbid ptsd +dep &
16 & 14 & 14 & 0 & 3+ forms experienced & negative\tabularnewline
38 & incomplete schooling & 2 & Yes & 10 & 7 & 16 & depressed / PTSD seperately & 26 & 7 & 10 & 1 & 2 forms experienced forms experienced form only & positive\tabularnewline
28 & matric/post school qualification & 3 & Yes & 11 & 8 & 15 & depressed / PTSD seperately & 26 & 15 & 15 & 1 & 2 forms experienced &
negative\tabularnewline
30 & incomplete schooling & 0 & Yes & 0 & 9 & 9 & depressed / PTSD seperately & 26 & 9 & 9 & 0 & 3+ forms experienced & negative\tabularnewline
38 & incomplete schooling & 1 & Yes & 0 & 7 & 11 & depressed / PTSD seperately & 30 & 12 & 12 & 0 & 3+ forms experienced & positive\tabularnewline
26 & incomplete schooling & 2 & Yes & 13 & 6 & 12 & no MH & 24 & 3 & 6 &
1 & 3+ forms experienced & negative\tabularnewline
33 & incomplete schooling & 0 & No & 0 & 8 & 12 & depressed / PTSD seperately & 26 & 12 & 15 & 0 & 3+ forms experienced & negative\tabularnewline
30 & matric/post school qualification & 1 & Yes & 7 & 6 & 14 & no MH &
27 & 4 & 7 & 0 & no violence & positive\tabularnewline
37 & incomplete schooling & 3 & Yes & 4 & 7 & 11 & depressed / PTSD seperately & 36 & 12 & 12 & 1 & 2 forms experienced forms

experienced
form only & positive\tabularnewline
30 & incomplete schooling & 1 & Yes & 3 & 10 & 15 & comorbid ptsd
+dep &
25 & 12 & 12 & 0 & 3+ forms experienced & positive\tabularnewline
29 & incomplete schooling & 0 & No & 13 & 6 & 13 & depressed / PTSD
seperately & 18 & 15 & 18 & 0 & 2 forms experienced forms
experienced
form only & negative\tabularnewline
46 & incomplete schooling & 5 & No & 9 & 6 & 18 & depressed / PTSD
seperately & 42 & 14 & 14 & 0 & 2 forms experienced &
negative\tabularnewline
20 & incomplete schooling & 0 & Yes & 0 & 8 & 12 & comorbid ptsd
+dep &
17 & 7 & 10 & 0 & 3+ forms experienced & negative\tabularnewline
33 & incomplete schooling & 2 & Yes & 0 & 9 & 10 & comorbid ptsd
+dep &
30 & 12 & 12 & 0 & 2 forms experienced & positive\tabularnewline
20 & incomplete schooling & 1 & Yes & 11 & 8 & 22 & depressed / PTSD
seperately & 23 & 11 & 14 & 0 & 2 forms experienced &
positive\tabularnewline
23 & matric/post school qualification & 2 & Yes & 13 & 6 & 11 & no
MH &
17 & 8 & 7 & 0 & 2 forms experienced forms experienced form only &
negative\tabularnewline
46 & incomplete schooling & 2 & No & 7 & 9 & 15 & depressed / PTSD
seperately & 15 & 13 & 16 & 0 & 3+ forms experienced &
positive\tabularnewline
35 & matric/post school qualification & 1 & Yes & 9 & 8 & 11 &
depressed
/ PTSD seperately & 33 & 16 & 19 & 0 & 2 forms experienced &
negative\tabularnewline
31 & incomplete schooling & 0 & Yes & 6 & 8 & 19 & depressed / PTSD
seperately & 29 & 4 & 7 & 0 & 2 forms experienced &
positive\tabularnewline
59 & incomplete schooling & 2 & No & 4 & 6 & 13 & depressed / PTSD
seperately & 26 & 7 & 7 & 1 & 3+ forms experienced &
positive\tabularnewline
29 & matric/post school qualification & 2 & Yes & 14 & 8 & 16 &
depressed / PTSD seperately & 27 & 12 & 12 & 0 & no violence &
negative\tabularnewline
24 & matric/post school qualification & 0 & Yes & 11 & 7 & 13 &
comorbid
ptsd +dep & 20 & 4 & 7 & 0 & no violence & negative\tabularnewline
36 & incomplete schooling & 3 & No & 12 & 9 & 14 & comorbid ptsd
+dep &
34 & 9 & 9 & 0 & 2 forms experienced forms experienced form only &
negative\tabularnewline
30 & incomplete schooling & 1 & Yes & 0 & 6 & 12 & no MH & 27 & 6 &
6 &
0 & 2 forms experienced forms experienced form only &
negative\tabularnewline
31 & matric/post school qualification & 5 & Yes & 9 & 9 & 9 & no MH
& 27

& 7 & 10 & 0 & 2 forms experienced & positive\tabularnewline
25 & matric/post school qualification & 1 & Yes & 0 & 7 & 9 & comorbid
ptsd +dep & 22 & 11 & 14 & 0 & no violence & positive\tabularnewline
22 & matric/post school qualification & 0 & Yes & 11 & 8 & 21 & no MH &
20 & 13 & 13 & 0 & 2 forms experienced & negative\tabularnewline
35 & incomplete schooling & 3 & Yes & 8 & 9 & 11 & comorbid ptsd +dep &
31 & 7 & 7 & 0 & 2 forms experienced & negative\tabularnewline
35 & incomplete schooling & 3 & Yes & 8 & 6 & 18 & depressed / PTSD seperately & 30 & 11 & 11 & 0 & 2 forms experienced forms experienced
form only & positive\tabularnewline
43 & incomplete schooling & 3 & Yes & 6 & 9 & 15 & comorbid ptsd +dep &
23 & 9 & 12 & 0 & 3+ forms experienced & positive\tabularnewline
30 & incomplete schooling & 2 & No & 13 & 6 & 16 & comorbid ptsd +dep &
20 & 18 & 18 & 0 & 2 forms experienced & negative\tabularnewline
31 & matric/post school qualification & 1 & Yes & 14 & 6 & 13 & no MH &
30 & 13 & 13 & 0 & 2 forms experienced forms experienced form only & negative\tabularnewline
32 & incomplete schooling & 2 & No & 10 & 6 & 13 & depressed / PTSD seperately & 30 & 13 & 13 & 0 & 2 forms experienced forms experienced
form only & negative\tabularnewline
27 & incomplete schooling & 1 & No & 12 & 7 & 15 & depressed / PTSD seperately & 23 & 12 & 12 & 1 & 2 forms experienced forms experienced
form only & negative\tabularnewline
26 & incomplete schooling & 3 & No & 10 & 8 & 13 & comorbid ptsd +dep &
21 & 11 & 11 & 0 & 2 forms experienced & positive\tabularnewline
20 & incomplete schooling & 0 & Yes & 10 & 8 & 16 & comorbid ptsd +dep &
17 & 14 & 13 & 0 & no violence & positive\tabularnewline
43 & matric/post school qualification & 6 & Yes & 12 & 22 & 24 & comorbid ptsd +dep & 41 & 15 & 15 & 0 & 3+ forms experienced & negative\tabularnewline
19 & incomplete schooling & 1 & Yes & 10 & 7 & 19 & no MH & 17 & 11 & 14
& 0 & 2 forms experienced forms experienced form only & negative\tabularnewline
37 & incomplete schooling & 2 & Yes & 11 & 6 & 8 & no MH & 33 & 5 & 8 &
0 & 2 forms experienced forms experienced form only & positive\tabularnewline
24 & incomplete schooling & 2 & No & 11 & 13 & 16 & no MH & 21 & 17 & 17
& 0 & 3+ forms experienced & negative\tabularnewline
46 & matric/post school qualification & 2 & Yes & 12 & 12 & 22 & depressed / PTSD seperately & 39 & 13 & 16 & 0 & 3+ forms

experienced &
positive\tabularnewline
32 & incomplete schooling & 4 & No & 7 & 7 & 11 & no MH & 30 & 6 & 6
& 0
& 2 forms experienced forms experienced form only &
negative\tabularnewline
37 & incomplete schooling & 4 & Yes & 7 & 9 & 12 & depressed / PTSD
seperately & 25 & 15 & 18 & 0 & 2 forms experienced forms
experienced
form only & positive\tabularnewline
38 & incomplete schooling & 3 & Yes & 10 & 8 & 25 & comorbid ptsd
+dep &
26 & 11 & 11 & 1 & 3+ forms experienced & positive\tabularnewline
24 & incomplete schooling & 1 & Yes & 11 & 6 & 16 & depressed / PTSD
seperately & 23 & 11 & 11 & 0 & 2 forms experienced forms
experienced
form only & negative\tabularnewline
39 & incomplete schooling & 2 & Yes & 5 & 7 & 10 & comorbid ptsd
+dep &
34 & 7 & 7 & 0 & 2 forms experienced & positive\tabularnewline
27 & incomplete schooling & 3 & No & 16 & 6 & 13 & no MH & 25 & 9 &
12 &
0 & 2 forms experienced & positive\tabularnewline
23 & incomplete schooling & 1 & Yes & 12 & 9 & 12 & comorbid ptsd
+dep &
23 & 6 & 9 & 0 & 3+ forms experienced & negative\tabularnewline
21 & matric/post school qualification & 1 & No & 10 & 6 & 16 &
depressed
/ PTSD seperately & 19 & 6 & 6 & 0 & 3+ forms experienced &
negative\tabularnewline
23 & incomplete schooling & 2 & No & 15 & 10 & 17 & depressed / PTSD
seperately & 18 & 13 & 12 & 0 & 3+ forms experienced &
positive\tabularnewline
28 & matric/post school qualification & 1 & Yes & 0 & 8 & 12 &
comorbid
ptsd +dep & 18 & 9 & 9 & 0 & 3+ forms experienced &
negative\tabularnewline
23 & incomplete schooling & 0 & Yes & 12 & 6 & 15 & no MH & 20 & 4 &
3 &
0 & 2 forms experienced & positive\tabularnewline
31 & matric/post school qualification & 2 & No & 0 & 10 & 20 &
comorbid
ptsd +dep & 27 & 19 & 19 & 1 & 3+ forms experienced &
positive\tabularnewline
40 & incomplete schooling & 6 & Yes & 11 & 9 & 18 & no MH & 35 & 4 &
7 &
0 & 2 forms experienced & negative\tabularnewline
23 & incomplete schooling & 1 & Yes & 0 & 7 & 12 & comorbid ptsd
+dep &
20 & 8 & 8 & 0 & 2 forms experienced & positive\tabularnewline
40 & matric/post school qualification & 2 & No & 9 & 6 & 11 &
comorbid
ptsd +dep & 37 & 8 & 11 & 0 & 2 forms experienced forms experienced
form

only & negative\tabularnewline
40 & incomplete schooling & 2 & Yes & 13 & 8 & 16 & comorbid ptsd +dep &
39 & 15 & 15 & 0 & 2 forms experienced & positive\tabularnewline
36 & incomplete schooling & 4 & Yes & 11 & 6 & 16 & comorbid ptsd +dep &
35 & 9 & 9 & 0 & no violence & positive\tabularnewline
34 & incomplete schooling & 4 & Yes & 14 & 12 & 23 & depressed / PTSD
seperately & 30 & 16 & 16 & 0 & 3+ forms experienced & positive\tabularnewline
25 & matric/post school qualification & 0 & Yes & 6 & 6 & 22 & no MH &
21 & 4 & 7 & 0 & 3+ forms experienced & negative\tabularnewline
33 & incomplete schooling & 3 & No & 12 & 6 & 13 & no MH & 21 & 13 & 13
& 0 & 2 forms experienced forms experienced form only & positive\tabularnewline
41 & incomplete schooling & 4 & No & 11 & 9 & 11 & depressed / PTSD
seperately & 34 & 2 & 5 & 0 & 3+ forms experienced & positive\tabularnewline
22 & matric/post school qualification & 0 & No & 0 & 7 & 16 & depressed
/ PTSD seperately & 20 & 12 & 15 & 0 & 3+ forms experienced & negative\tabularnewline
38 & incomplete schooling & 1 & No & 6 & 6 & 14 & comorbid ptsd +dep &
24 & 16 & 16 & 0 & 3+ forms experienced & positive\tabularnewline
30 & matric/post school qualification & 1 & Yes & 3 & 6 & 10 & no MH &
26 & 3 & 6 & 0 & no violence & positive\tabularnewline
29 & matric/post school qualification & 2 & Yes & 13 & 7 & 15 & comorbid
ptsd +dep & 27 & 12 & 12 & 0 & 2 forms experienced & negative\tabularnewline
41 & incomplete schooling & 3 & Yes & 12 & 6 & 13 & no MH & 39 & 14 & 14
& 0 & 2 forms experienced forms experienced form only & negative\tabularnewline
25 & incomplete schooling & 1 & Yes & 12 & 10 & 15 & depressed / PTSD
seperately & 20 & 12 & 12 & 0 & 2 forms experienced forms experienced
form only & negative\tabularnewline
33 & incomplete schooling & 1 & Yes & 11 & 6 & 13 & comorbid ptsd +dep &
30 & 14 & 17 & 0 & 2 forms experienced forms experienced form only & positive\tabularnewline
32 & incomplete schooling & 2 & Yes & 10 & 7 & 16 & no MH & 30 & 12 & 12
& 0 & 2 forms experienced forms experienced form only & positive\tabularnewline
36 & incomplete schooling & 3 & No & 11 & 7 & 16 & depressed / PTSD
seperately & 31 & 4 & 7 & 0 & 2 forms experienced forms experienced

form
only & negative\tabularnewline
29 & incomplete schooling & 2 & No & 7 & 6 & 13 & depressed / PTSD
seperately & 27 & 11 & 11 & 0 & 2 forms experienced &
negative\tabularnewline
18 & incomplete schooling & 0 & No & 0 & 6 & 13 & no MH & 16 & 10 &
13 &
0 & 2 forms experienced forms experienced form only &
negative\tabularnewline
28 & incomplete schooling & 1 & Yes & 9 & 6 & 13 & comorbid ptsd
+dep &
26 & 8 & 11 & 0 & 2 forms experienced & positive\tabularnewline
28 & incomplete schooling & 1 & Yes & 15 & 8 & 14 & depressed / PTSD
seperately & 26 & 12 & 12 & 0 & 2 forms experienced forms
experienced
form only & positive\tabularnewline
24 & incomplete schooling & 0 & Yes & 13 & 11 & 15 & comorbid ptsd
+dep
& 15 & 15 & 15 & 0 & 3+ forms experienced & negative\tabularnewline
32 & matric/post school qualification & 1 & Yes & 11 & 9 & 13 &
depressed / PTSD seperately & 30 & 12 & 15 & 0 & no violence &
negative\tabularnewline
20 & incomplete schooling & 0 & No & 12 & 8 & 16 & comorbid ptsd
+dep &
18 & 12 & 12 & 0 & no violence & negative\tabularnewline
36 & incomplete schooling & 3 & Yes & 4 & 7 & 17 & depressed / PTSD
seperately & 33 & 8 & 11 & 0 & 2 forms experienced forms experienced
form only & positive\tabularnewline
45 & matric/post school qualification & 3 & Yes & 0 & 7 & 19 & no MH
&
35 & 12 & 12 & 0 & 2 forms experienced & positive\tabularnewline
26 & incomplete schooling & 1 & Yes & 6 & 8 & 12 & depressed / PTSD
seperately & 21 & 10 & 10 & 1 & 3+ forms experienced &
negative\tabularnewline
22 & incomplete schooling & 1 & Yes & 12 & 7 & 18 & depressed / PTSD
seperately & 22 & 6 & 6 & 1 & 2 forms experienced &
negative\tabularnewline
27 & incomplete schooling & 1 & Yes & 4 & 7 & 13 & comorbid ptsd
+dep &
20 & 8 & 11 & 1 & 3+ forms experienced & negative\tabularnewline
22 & matric/post school qualification & 1 & Yes & 5 & 6 & 15 & no MH
&
20 & 5 & 8 & 0 & no violence & negative\tabularnewline
29 & incomplete schooling & 1 & No & 7 & 6 & 13 & comorbid ptsd +dep
&
29 & 13 & 13 & 0 & no violence & negative\tabularnewline
34 & incomplete schooling & 2 & Yes & 0 & 6 & 14 & comorbid ptsd
+dep &
24 & 12 & 12 & 0 & 2 forms experienced forms experienced form only &
negative\tabularnewline
20 & incomplete schooling & 2 & Yes & 6 & 8 & 14 & depressed / PTSD
seperately & 14 & 14 & 14 & 0 & 2 forms experienced forms
experienced
form only & negative\tabularnewline

```
26 & matric/post school qualification & 1 & Yes & 12 & 8 & 14 &
depressed / PTSD seperately & 19 & 11 & 11 & 0 & 2 forms experienced
forms experienced form only & negative\tabularnewline
27 & incomplete schooling & 3 & No & 15 & 9 & 14 & depressed / PTSD
seperately & 24 & 7 & 10 & 0 & 3+ forms experienced &
positive\tabularnewline
22 & matric/post school qualification & 1 & Yes & 0 & 8 & 17 &
depressed
/ PTSD seperately & 19 & 8 & 11 & 0 & 2 forms experienced forms
experienced form only & negative\tabularnewline
42 & incomplete schooling & 1 & Yes & 6 & 12 & 21 & no MH & 34 & 5 &
8 &
0 & 2 forms experienced & positive\tabularnewline
24 & incomplete schooling & 2 & Yes & 14 & 7 & 21 & depressed / PTSD
seperately & 18 & 8 & 8 & 0 & 3+ forms experienced &
positive\tabularnewline
27 & incomplete schooling & 3 & No & 11 & 7 & 13 & depressed / PTSD
seperately & 26 & 9 & 8 & 0 & 2 forms experienced &
positive\tabularnewline
20 & matric/post school qualification & 1 & Yes & 7 & 6 & 17 &
comorbid
ptsd +dep & 19 & 3 & 6 & 0 & 3+ forms experienced &
negative\tabularnewline
37 & incomplete schooling & 1 & Yes & 11 & 8 & 15 & comorbid ptsd
+dep &
35 & 3 & 6 & 0 & 2 forms experienced & positive\tabularnewline
21 & matric/post school qualification & 1 & Yes & 10 & 6 & 16 &
comorbid
ptsd +dep & 20 & & & 0 & 2 forms experienced forms experienced form
only
& negative\tabularnewline
31 & incomplete schooling & 0 & Yes & 6 & 9 & 14 & comorbid ptsd
+dep &
25 & 14 & 17 & 0 & 3+ forms experienced & negative\tabularnewline
27 & matric/post school qualification & 2 & Yes & 4 & 7 & 11 &
comorbid
ptsd +dep & 23 & 7 & 7 & 0 & 2 forms experienced &
positive\tabularnewline
33 & incomplete schooling & 2 & Yes & 11 & 7 & 16 & depressed / PTSD
seperately & 28 & 12 & 12 & 0 & 2 forms experienced &
negative\tabularnewline
22 & incomplete schooling & 1 & Yes & 5 & 7 & 18 & no MH & 19 & 11 &
11
& 1 & no violence & negative\tabularnewline
\bottomrule
\end{longtable}
```
